# A Clearance Period after Soluble Lead Nanoparticle Inhalation Did Not Ameliorate the Negative Effects on Target Tissues Due to Decreased Immune Response

**DOI:** 10.3390/ijms21228738

**Published:** 2020-11-19

**Authors:** Jana Dumková, Tereza Smutná, Lucie Vrlíková, Bohumil Dočekal, Daniela Kristeková, Zbyněk Večeřa, Zuzana Husáková, Veronika Jakešová, Adriena Jedličková, Pavel Mikuška, Lukáš Alexa, Pavel Coufalík, Michaela Tvrdoňová, Kamil Křůmal, Tomáš Vaculovič, Viktor Kanický, Aleš Hampl, Marcela Buchtová

**Affiliations:** 1 Department of Histology and Embryology, Faculty of Medicine, Masaryk University, 625 00 Brno, Czech Republic; jdumkova@med.muni.cz (J.D.); terka.smutna12@gmail.com (T.S.); ahampl@med.muni.cz (A.H.); 2 Laboratory of Molecular Morphogenesis, Institute of Animal Physiology and Genetics, v.v.i., Czech Academy of Sciences, 602 00 Brno, Czech Republic; vrlikova@iach.cz (L.V.); daniela.kristekova@gmail.com (D.K.); veronikam@volny.cz (V.J.); adri.jedlickova@gmail.com (A.J.); 3 Department of Environmental Analytical Chemistry, Institute of Analytical Chemistry, v.v.i., Czech Academy of Sciences, 602 00 Brno, Czech Republic; docekal@iach.cz (B.D.); vecera@iach.cz (Z.V.); mikuska@iach.cz (P.M.); alexa@iach.cz (L.A.); coufalik@iach.cz (P.C.); krumal@iach.cz (K.K.); 4 Section of Animal Physiology and Immunology, Department of Experimental Biology, Faculty of Science, Masaryk University, 625 00 Brno, Czech Republic; 5 Department of Chemistry, Faculty of Science, Masaryk University, 625 00 Brno, Czech Republic; zuzka.husakova@seznam.cz (Z.H.); 358018@mail.muni.cz (M.T.); vaca_777@yahoo.com (T.V.); viktork@chemi.muni.cz (V.K.)

**Keywords:** clearance, lead nanoparticles, inhalation, LA-ICP-MS imaging, toxicity

## Abstract

The inhalation of metal (including lead) nanoparticles poses a real health issue to people and animals living in polluted and/or industrial areas. In this study, we exposed mice to lead(II) nitrate nanoparticles [Pb(NO_3_)_2_ NPs], which represent a highly soluble form of lead, by inhalation. We aimed to uncover the effects of their exposure on individual target organs and to reveal potential variability in the lead clearance. We examined (i) lead biodistribution in target organs using laser ablation and inductively coupled plasma mass spectrometry (LA-ICP-MS) and atomic absorption spectrometry (AAS), (ii) lead effect on histopathological changes and immune cells response in secondary target organs and (iii) the clearance ability of target organs. In the lungs and liver, Pb(NO_3_)_2_ NP inhalation induced serious structural changes and their damage was present even after a 5-week clearance period despite the lead having been almost completely eliminated from the tissues. The numbers of macrophages significantly decreased after 11-week Pb(NO_3_)_2_ NP inhalation; conversely, abundance of alpha-smooth muscle actin (α-SMA)-positive cells, which are responsible for augmented collagen production, increased in both tissues. Moreover, the expression of nuclear factor κB (*NF-κB*) and selected cytokines, such as tumor necrosis factor alpha (*TNFα*), transforming growth factor beta 1 (*TGFβ1*), interleukin 6(*IL-6*), *IL-1α* and *IL-1β* , displayed a tissue-specific response to lead exposure. In summary, diminished inflammatory response in tissues after Pb(NO_3_)_2_ NPs inhalation was associated with prolonged negative effect of lead on tissues, as demonstrated by sustained pathological changes in target organs, even after long clearance period.

## 1. Introduction

Ambient airborne particulate matter (PM) is considered as an important environmental pollutant with adverse effect on human health [1]. However, recent studies indicated that the adverse health effects of PM cannot be solely explained by the mass concentration of PM, but the size of particulates and their chemical composition play also an important role [2,3,4].

Ambient PM is a very complex multi-component mixture of organic material and inorganic compounds, including ionic species, crustal elements and metals [3,4,5]. Metals are present in the PM both in insoluble form as oxides or mineralized species [6], and partly soluble forming a dissolved metal fraction [5]. The water-soluble fraction of the metal was recently found to be responsible for acute respiratory illnesses and child asthma [7,8,9].

Pb is one of the most abundant metals in aerosols [10,11,12,13,14]. The limit for airborne Pb of 0.5 µg/m^3^ determined by the World Health Organization is regularly exceeded in many cities around the world when the measured concentrations of airborne Pb in some areas have reached 20 µg/m^3^ in urban background sites and even more than 70 µg/m^3^ in industrial areas [13]. Recent studies indicate that a substantial fraction (≥35%) of total Pb content is soluble in water [5,11,15].

Due to their size, inhaled nanoparticles (NPs) can invade the lower respiratory tract more easily than corresponding larger forms and they are predominantly deposited in the alveolar regions of lungs. Although the inhalation of metal NPs, including those that contain Pb, poses a real threat to humans, only a few studies have evaluated the potential toxicity of Pb in different nanoforms [16,17,18,19]. Different approaches have been used for the applications of metal nano-suspensions into animal models, such as intraperitoneal injections [17], intravenous injections [18,20], intratracheal instillation [21], or by using specific inhalation devices for rodents [22] to study systemic intoxication.

Pb NPs in the air can come into contact with various parts of the body, such as the respiratory system, the olfactory region, the skin, the hair or the conjunctiva of the eyes. The animals also take care of their fur and therefore oral exposure to inhaled NPs can be higher than in humans. However, for in both animals and humans, the inhaled NPs exhibit extrapulmonary effects because of the clearance from the lungs through mucociliary transport [23]. Here, we exposed animals to Pb NPs in whole-body inhalation chambers to simulate natural conditions. We used very low Pb NP concentrations in inhaled air to reflect complex real situations of how humans are exposed to metal NPs in the ambient atmosphere. In our previous studies [19,24], we exposed the animals to lead oxide nanoparticles (PbO NPs), which is a poorly soluble Pb compound. Shortly, after subchronic PbONP inhalation, morphological alterations and tissue damage were observed mainly in the lungs as a primary target of the inhaled PbO NPs. After 5-week clearance period, the decreased level of pathological changes in the lungs was observed in all analyzed animals. Similarly, in the liver, a 5-week clearance period after 6-week PbONP inhalation illustrated the ability of this organ to effectively react to an increased lead load during the time of the experiment. Thereafter, clearance of ionic lead and PbONPs (Pb/PbONPs) from the lungs and liver was effective. In this study, we exposed the animals to lead(II) nitrate nanoparticles [Pb(NO_3_)_2_ NPs] to mimic the effect of the soluble form of Pb in PM. Lead(II) nitrate was selected because of its high solubility in water. Nitrate, the second part of the compound, is very important ionic component of PM [12]; however, its toxicity under normal concentration is low. Nitrate’s toxicity results from its conversion to nitrite in the body that at high concentration may cause methemoglobinemia [25].

A mass concentration of Pb(NO_3_)_2_ NPs of 68.6 μg/m^3^ (i.e., 42.9 μg Pb/m^3^), used for exposure of animals, corresponds to the exposure of city inhabitants during pollution peaks. Occupational Safety and Health Administration (OSHA) has set a permissible exposure limit for lead in the workplace air of 50 μg/m^3^.

We examined the Pb biodistribution, toxicological effects and clearance ability of target organs (lungs, liver, kidney, spleen, bone and blood) following sub-chronic inhalation of Pb(NO_3_)_2_ NPs using female mice CD-1 (ICR) line as a model organism. Our study revealed diminished inflammatory response in lungs and liver after 11-week Pb(NO_3_)_2_ NP inhalation, which was associated with prolonged negative effect of lead on tissues even after a 5-week clearance period. Therefore, highly soluble Pb(NO_3_)_2_ NPs evoke distinct effects on target organs than those observed for less soluble PbO NPs used in our previous study [24].

## 2. Results and Discussion

There are three typical routes—food, water and air [26]—of human exposure to metal contaminants. An approximate estimate of individual inputs for Pb is 20.5% from diet, 11.9% from drinking water, 43.7% from outdoor soil/dust, 23.7% from indoor dust and 0.1% from air by inhalation [27]. However, resuspended soil can be a significant source of atmospheric Pb [28]. Given that approximately 8–10% of Pb in adults and 40–50% in infants is absorbed through ingestion, compared up to 60% absorption through respiration, the inhalation pathway of this metal has recently gained attention [29,30]. It was predicted that 1 µg Pb/m^3^ in the air directly contributes 19 µg Pb/L blood in children (or 16 µg Pb/L blood in adults), indirectly (as air Pb increases Pb uptake by indirect pathways) up to 50 µg Pb/L blood (=5 µg Pb/dL) [31].

**Figure 1:**
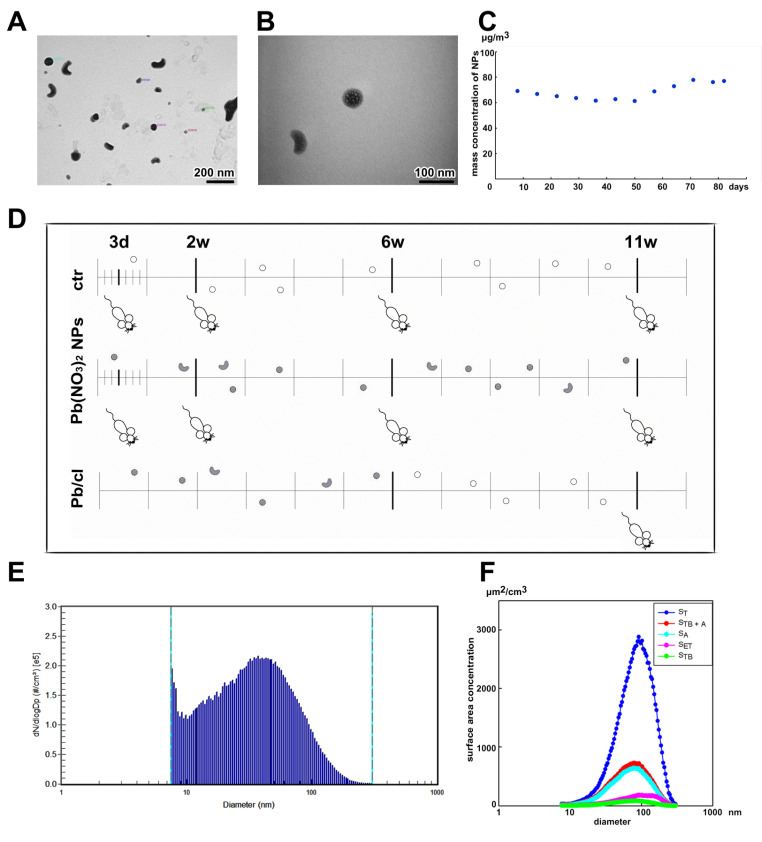
The characterization of Pb(NO_3_)_2_ nanoparticles (NPs) in the inhalation chambers, and the scheme of overall experimental design. (**A**,**B**) The characterization of Pb(NO_3_)_2_ NPs analyzed in the scanning transmission electron microscope (STEM). (**C**) Temporal trend of the mass concentration of Pb(NO_3_)_2_ NPs throughout the inhalation experiment. (**D**) Design of the inhalation experiment. The groups of animals inhaled clean air for a period of up to 11 weeks (ctr), the other groups inhaled air with Pb(NO_3_)_2_ NPs for the same period (Pb(NO_3_)_2_ NP), and one group inhaled air with Pb(NO_3_)_2_ NPs for 6 weeks and thereafter five weeks of clean air (clearance group—Pb/cl). Symbols of light circles indicate clean air, and symbols of dark circles or dark beans indicate NPs. (**E**) Particle number-size distribution of Pb(NO_3_)_2_ NPs in the inhalation chambers measured by Scanning Mobility Particle Sizer (SMPS). (**F**) Surface area of Pb(NO_3_)_2_ NPs size distribution (dS/dLogD_p_). The surface area of fractions of Pb(NO_3_)_2_ NPs deposited in the extrathoracic (S_ET_), tracheobronchial (S_TB_) and alveolar region (S_A_) of lungs, S_T_—the total surface area of generated Pb(NO_3_)_2_ NPs, S_TB+A_—the lung-deposited surface area.

For this reason, we selected NP inhalation in whole-body chambers as the delivery method that represents the most typical exposure condition in humans. Specifically, we tested the effect of sub-chronic (11 weeks) inhalation of Pb(NO_3_)_2_ NPs dispersed in the air (Figure 1, Table 1). First, we analyzed the influence of these highly soluble NPs on animals with a focus on their behavior inside living organisms, with mapping the ability of individual organs to eliminate the Pb burden after a clearance period.

Adult female mice divided into control (air) and exposed [Pb(NO_3_)_2_ NPs] groups were placed into inhalation chambers, where they were exposed to modified air for the entire experimental period. For each designated time point (3 days, 2 weeks, 6 weeks and 11 weeks), 10 biological replicates were evaluated in both control and Pb(NO_3_)_2_ NP-exposed groups (Figure 1). In addition, one group of mice inhaled air with Pb(NO_3_)_2_ NPs for 6 weeks, and thereafter clean air for 5 weeks (this group is further referred to as the clearance group: Pb/cl). Thus, at the end of the inhalation period (11 weeks), 10 biological replicates of control group, each Pb(NO_3_)_2_ NP–exposed group and the Pb/cl group were examined in the same way as the previously described experimental groups (Figure 1D).

To assess the toxicological impacts of NPs and their possible risks to human health, information about NP deposition in different human lung regions is required. Therefore, we simulated the deposition of inhaled Pb(NO_3_)_2_ NPs in different parts of the human respiratory tract, using deposition fractions calculated by the International Commission on Radiological Protection (ICRP) deposition model for the extrathoracic, tracheobronchial and alveolar regions [32]. The total surface area of generated Pb(NO_3_)_2_ NPs (i.e., 1.68 × 10^3^ µm^2^/cm^3^; S_T_) was calculated by SMPS spectrometer software from the measured particle size distribution. Based on the surface area of the NPs deposited in the extrathoracic region (S_ET_; the anterior and posterior nasal passage, larynx, pharynx and mouth), tracheobronchial region (S_TB_; trachea, bronchi and bronchioles) and alveolar region (S_A_; respiratory bronchioles and alveolar ducts), the highest Pb(NO_3_)_2_ NP surface area (25.3% of the total) is deposited in alveolar region of lungs, followed by the extrathoracic (7.4%) and tracheobronchial (3.3%) regions (Figure 1F). The deposition of NPs in human respiratory tract was compared with the deposition in mouse respiratory tract calculated using a MPPD model [33,34,35] for monodisperse aerosol with geometric mean diameter of 31.3 nm and mass concentration of 68.6 µg/m^3^. The depositions calculated using the MPPD model for the extrathoracic, tracheobronchial and alveolar regions of mouse respiratory tract (i.e., 33.2%, 6.69% and 12.8%, respectively) are different from those calculated by the ICRP model for human respiratory tract, which is probably caused mainly by different morphometry (i.e., mouse × human) and different aerosol parameters (i.e., polydisperse aerosol in ICRP model × monodisperse aerosol in the MPPD model) used for the calculation in both models.

Lung-deposited surface area (LDSA) concentration, another relevant metric for health effects of aerosol particles [36], combines the surface area of the NPs deposited in the alveolar and tracheobronchial regions. Therefore, we evaluated the particle size distribution of S_T_, S_ET_, S_TB_ and S_A_, as well as the LDSA (S_TB+A_) (Figure 1F). The LDSA corresponds to 28.6% of total surface area of prospectively inhaled Pb(NO_3_)_2_ NPs, while the sum of S_ET_, S_TB_ and S_A_ is 36.0% of total surface area of inhaled Pb(NO_3_)_2_ NPs (Figure 1F).

### 2.1. Pb Accumulates in All Analysed Target Organs upon Pb(NO_3_)_2_ NP Inhalation

In animals exposed to Pb(NO_3_)_2_ NPs, the Pb concentration in the organs continuously increased with the increasing exposure time (Table 2). After a 5-week clearance period, the Pb concentration in the lungs and spleen decreased to the limit of detection (LOD). This was not the case for the femur, kidney and liver, in which Pb was clearly detectable even after a 5-week clearance period.

It needs to be said that because of the technical reasons the Pb(NO_3_)_2_ NPs concentration in the inhaled air was not absolutely constant and fluctuated slightly during the experiment (Figure 1C). This fact was determined by collecting NPs on the filters and the evaluation of the mass concentration of Pb(NO_3_)_2_ that was lower after 6 weeks of Pb(NO_3_)_2_ NP exposure in comparison to the earlier timepoints (Figure 1C). Still, these small irregularities in the mass concentration of inhaled Pb(NO_3_)_2_ were not reflected by the total Pb concentrations detected in the individual organs and in the blood (Table 2 and Table 3), with the exception of kidneys where stagnation of Pb level between the 2nd week and 6th week timepoint was observed.

The response of individual organs studied here, in animals exposed to Pb(NO_3_)_2_, also slightly varied and caused the changes of their weight coefficients (Figure S1). The weight coefficients of organs were expressed as wet weight of organ (g)/dead body weight (g) × 100. After 2 weeks, the liver weight coefficient significantly increased (*p* < 0.001), while the kidney coefficient decreased (*p* < 0.05 for left; *p* < 0.01 for right) and the spleen coefficient decreased (*p* < 0.01) compared with control mice. After 11 weeks, there was a decrease in the liver weight coefficient in the Pb/cl compared with the control group (*p* < 0.05). Some contribution to organ weight variability could be due to organ dissection; however, we observed similar alterations of organ weight coefficients after metal NP exposure, as was described previously in other studies [37,38].

### 2.2. Inhalation of Pb(NO_3_)_2_ NPs Causes Pulmonary Injury

The lungs are a major portal for the entrance of inhaled particles. Metal NPs, including Pb nanoforms, can invade the lower respiratory passages down to alveolar region more easily than larger forms. First, we analyzed the presence of Pb in lungs after their exposure to Pb(NO_3_)_2_ NPs at different time points and found a continuous increase in Pb as the NP inhalation period increased (Figure S2, Table 2). A 5-week clearance period positively affected Pb clearance from the lungs; indeed, the Pb concentration decreased almost to the LOD (26 ng/g). Elemental imaging of lungs after Pb(NO_3_)_2_ NP inhalation revealed Pb being regularly distributed in all parts of the lungs; and after a 5-week clearance period, there was an obvious reduction in the Pb level (Figure S2).

**Figure 2:**
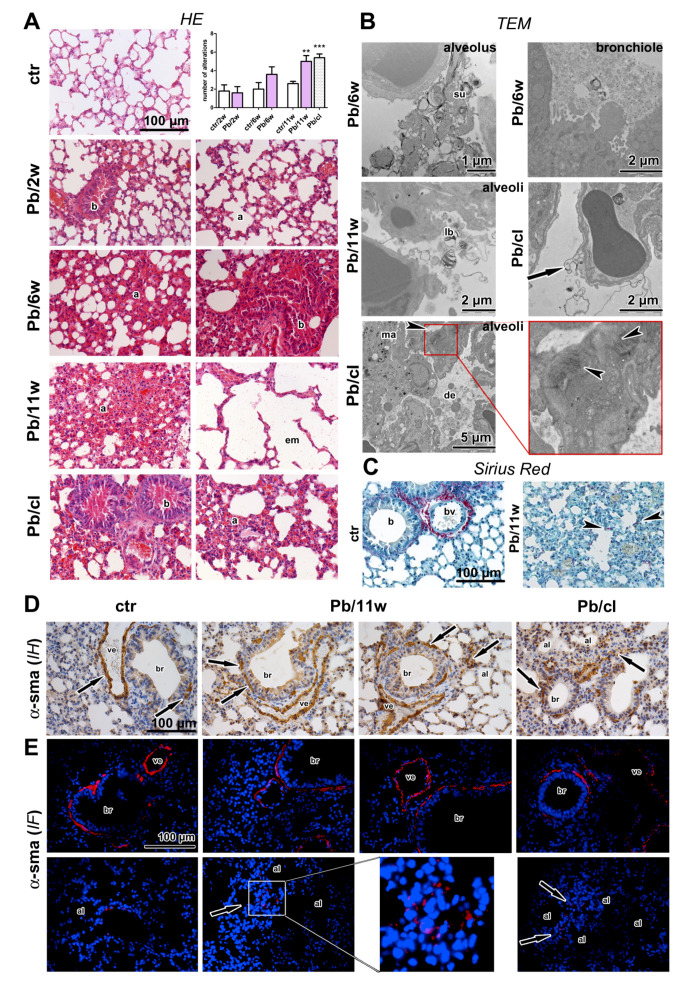
The effect of Pb(NO_3_)_2_ NP inhalation and its clearance on the lungs. (**A**) The lungs in control animals, and after 2-week Pb(NO_3_)_2_ NP inhalation were without serious pathological changes. The lungs after 6 and 11-week Pb(NO_3_)_2_ NP inhalation exhibited remodeling in bronchiolar (b), and alveolar areas (a). The serious damage of lung parenchyma with destroyed alveolar septs and alveolar emphysema detected in some areas (em). Alteration of lung tissue was still detected after a 5-week clearance period. Statistical evaluation of the histopathological changes in both control and Pb(NO_3_)_2_ NP-exposed groups in the experiments delineated in Table S1. The graphs values refer to average ± SD; ** *p* < 0.01, and *** *p* < 0.001 compared with the corresponding control group (ctr) by unpaired *t*-test. (**B**) The expressive lung secretion was found in bronchioles, and alveolar spaces by transmission electron microscopy analysis (TEM). The higher amount of surfactant (su), lamellar bodies (lb), cell detritus (de) and enormous macrophages (ma) were in the alveoli. Arrow points to the damaged membranes of alveolar cell, arrowheads indicate high amount of collagen fibrils in the alveolar septs. The nanoparticles were not present in lung tissue. (**C**) Collagen fibers (red) were around blood vessels (bv), and bronchioles (b) in controls. After 11-week Pb(NO_3_)_2_ NP inhalation, collagen fibers were in alveolar regions (arrowheads). (**D**) Immunohistochemical detection (IH) of α-SMA positive cells in the walls of bronchioles (br) or blood vessels (ve) in controls (arrows), and after 11-week of Pb(NO_3_)_2_ NP inhalation in the walls of bronchioles (br), and in the alveolar regions (al, arrows). (**E**) Immunofluorescent detection (IF) of α-SMA positive cells in the walls of bronchioles (br) or blood vessels (ve) in controls, Pb(NO_3_)_2_ NP and Pb/cl inhaled groups. α-SMA positive cells aggregated in some alveolar regions (al, arrows, in box) after Pb(NO_3_)_2_ NP inhalation. Scale bar in panels (**A**,**C**–**E**) = 100 µm.

**Figure 3:**
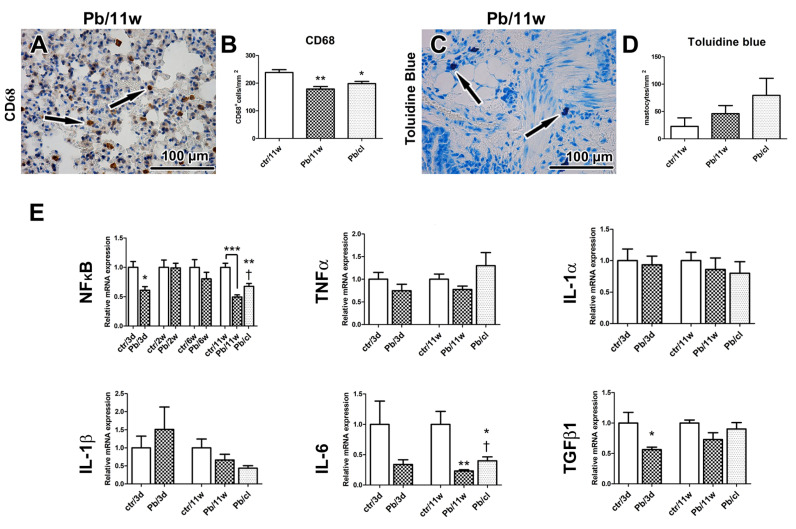
The effect of Pb(NO_3_)_2_ NP inhalation and its clearance on the lung inflammation. (**A**) Detection of CD68-positive cells (marker of macrophages) in lungs (arrows). (**B**) The number of macrophages was decreased in Pb(NO_3_)_2_ NPs and Pb/cl groups. The difference in number of CD68-positive cells was statistically significant compared with the control group. The graphs values indicate average ± SD; * *p* < 0.05, ** *p* < 0.01. (**C**) Detection of Toluidine Blue-positive cells (marker of mastocytes) in lungs (arrows). (**D**) The number of mastocytes was slightly increased in Pb(NO_3_)_2_ NP and Pb/cl groups. The graphs values indicate average ± SD. Scale bar in panels (**A**,**C**) = 100 µm. (**E**) Gene expression of *NF-κB* and cytokines at selected time points after Pb(NO_3_)_2_ NP inhalation. The graphs values indicate average ± SD; * *p* < 0.05, ** *p* < 0.01, *** *p* < 0.001 compared with the corresponding control group (ctr), and † *p* < 0.05 compared with the corresponding Pb(NO_3_)_2_ NP group by unpaired *t*-test.

Histological analyses revealed numerous signs of inflammation and focal destruction of lung parenchyma in animals exposed to Pb(NO_3_)_2_ NPs; the damage depended on the exposure time (Figure 2 and Figure 3, Table S1). These findings were similar to what has been described in the inhalation experiment with other metal NPs: PbO [19], titanium dioxide (TiO_2_) [39] and cobalt (Co) [40]. The damaged lung parenchyma included destroyed alveolar septa, with alveolar emphysema initiated in some areas. The lungs of exposed animals exhibited several signs of inflammation, such as hyperemia; thickened alveolar septa with increased cellularity; regions with peribronchial and perivascular lymphocyte infiltrates; and areas of atelectasis and focal bronchiolitis. All of these distinct histopathological changes in the lungs were evaluated and scored for individual groups (Table S1). The extent of histopathological changes in lungs was statistically significant after 11 weeks (*p* < 0.01).

While chemical and LA-ICP-MS detection documented the absence of Pb in the lungs after a 5-week clearance period, the histopathological analysis uncovered persistent inflammatory changes in the lungs (*p* < 0.001, Figure 2A, Table S1). The lungs of Pb/cl animals still exhibited several signs of inflammation: Hyperemia, thickened alveolar septa with increased cellularity, higher number of lung macrophages, regions with peribronchial and perivascular mononuclear infiltrates and focal bronchiolitis (Figure 2 and Figure 3).

We next performed ultrastructural analysis of lung tissue by transmission electron microscopy (TEM) to identify the localization of Pb(NO_3_)_2_ NPs in lungs (Figure 2B). There were no NPs in bronchioles or alveoli, which documented the rapid solubility of Pb(NO_3_)_2_ NPs during transport through respiratory passages. However, it is necessary to mention that we are only able to distinguish larger particles or aggregates in TEM; small single NPs cannot be unambiguously identified because of their similarity to ribosomes so that we cannot exclude their presence in pneumocytes using this morphological approach.

The lumens of bronchioles and alveoli contained inflammatory cells, cellular debris and accumulated surfactant; membranes of alveolar epithelial cells were severely damaged (Figure 2B). We also found majority of lamellar bodies being ejected from alveolar type II cells into the alveolar airspaces after 11-week Pb(NO_3_)_2_ NP inhalation (Figure 2B). It seems plausible that abundant surfactant seen by TEM may contribute to solubility of NPs in lower respiratory passages.

Collagen fibers were detected in the walls of blood vessels and bronchioles in the control animals. After 11-week Pb(NO_3_)_2_ NP inhalation, there were ectopic collagen fibers in the alveolar regions of lungs (Figure 2C). TEM also confirmed the presence of higher amount of collagen fibrils in the alveolar septa (Figure 2B). Augmented collagen production and induction of fibrosis can be associated with activation of myofibroblasts [41,42] and their expression of α-smooth muscle actin (α-SMA) [43]. Previous studies have reported the upregulation of α-SMA expression as a progressive fibrosis marker in the lungs after metal NP exposure [44,45]. We observed α-SMA-positive cells located in the walls of larger bronchioles or blood vessels in the control animals and Pb(NO_3_)_2_ NP inhalation increased their appearance in the walls of small bronchioles and aggregation in alveolar regions in the Pb(NO_3_)_2_ NP and Pb/cl groups (Figure 2D,E). Therefore, interstitial fibrosis was induced after 6-week of Pb(NO_3_)_2_ NP inhalation and was still active after a 5-week clearance period.

### 2.3. Inhalation of Pb(NO_3_)_2_ NPs Alters the Number of Lung Macrophages

Particles smaller than 0.5 µm can accumulate in the alveolar parts of the lung and induce an inflammatory response [21]. Although inflammation is usually protective, it can cause serious injury to the organ in the case of prolonged persistence. We found a reduced number of lung macrophages (detected as CD68-positive cells) after 11-week Pb(NO_3_)_2_ NP inhalation (*p* < 0.01, compared with the corresponding control group; Figure 3A,B, Figure S3, Table S2). A similar decrease of CD68-positive cells was noticed in Pb/cl animals (*p* < 0.05) compared with the corresponding control animals (Figure 3A,B, Table S2). Especially in lung infiltrates, the number of mastocytes (as visualized by Toluidine Blue) and the presence of neutrophils (as visualized by immunolabeling of myeloperoxidase, MPO) was increased (Figure 3, Figure S3). Taken together, soluble engineered Pb(NO_3_)_2_ NPs used at a very low concentration—corresponding to environmental levels of metal NPs—induced abnormalities in lung morphology that persisted even after a 5-week clearance period.

Next, we quantified immune system response on mRNA level. NF-κB is a family of transcription proteins that regulate genes involved in different processes of the immune responses [46]. NF-κB belongs to the first responders to variable harmful stimuli or to cytokines, such as tumor necrosis factor (TNF) or interleukin 1 (IL-1) [47]. Exposure to heavy metal compounds can inhibit binding of NF-κB to DNA and block this signaling pathway. Here, both 3-day and 11-week exposure to Pb(NO_3_)_2_ NP inhalation has led to significant downregulation of *NF-κB* in the lungs (Figure 3E) and the clearance period enabled for return of its expression close to its normal level (Figure 3E).

TNFα and IL-6 are major proinflammatory cytokines, while the IL-1 family includes 11 members expressed by numerous cell types, which comprises both proinflammatory and anti-inflammatory responses [48]. Previously, the exposure to NPs was found to deregulate many cytokines, including several IL family members or TNFα [49] and TNF signaling was previously linked to NF-κB activation [48]. Here, we observed downregulation of *TNFα* in the lungs after the shortest (3 days) and the longest (11 weeks) exposures (Figure 3E). Both *IL-1α* and *IL-1β* were downregulated in the lungs after the longest (11 weeks) exposure to Pb(NO_3_)_2_ NPs (Figure 3E) with both *IL-1* mRNA levels becoming even lower after a 5-week clearance period (Figure 3E).

IL-6 is produced by a wide range of cells, including macrophages or hepatocytes in response to tissue injuries [50]. While the IL-6 trans-signaling pathway is rather pro-inflammatory, the classic IL-6 signaling through the membrane-bound receptor is mainly involved in regenerative processes [51]. In the lungs, *IL-6* was downregulated after the shortest (3 days) and the longest (11 weeks) (*p* < 0.01) exposures to Pb(NO_3_)_2_ NPs. A 5-week clearance then has led to upregulation of *IL-6* mRNA in the lungs (*p* < 0.05 compared with the 11-week exposure).

TGFβ1 is an inflammatory cytokine secreted predominantly by leukocytes but also by other cell types, including macrophages [48]. In some conditions, its effect may increase the risk of pathologic fibrosis so that TGFβ1 is considered to be a potent profibrotic mediator [41]. However, our study did not confirm the increased level of this profibrotic cytokine after Pb(NO_3_)_2_ NP inhalation maybe due to mild alteration of lung parenchyma.

Activated macrophages are divided into two subpopulations: Classical (M1, pro-inflammatory) that predominantly produce IL-1β, IL-5 and TNFα; and alternative (M2, anti-inflammatory) that produce TGFβ1, vascular endothelial growth factor (VEGF), IL-10 and IL-12 [52]. Thus, TNFα, and TGFβ are cytokines produced by different population of macrophages with distinct final effects. In our experimental conditions, the shortest (3 days) and the longest (11 weeks) exposures to Pb(NO_3_)_2_ NPs were here associated with significant downregulation in *TGFβ1* mRNA in the lungs (*p* < 0.05) compared with the corresponding control animals (Figure 3E). The 5-week clearance allowed for some return of the *TGFβ1* mRNA towards its original level, when compared to the corresponding Pb(NO_3_)_2_ NP-exposed animals. Our findings on the effects of Pb(NO_3_)_2_ NPs on *TNFα* and *TGFβ* mRNA levels are similar to those seen in animals exposed to other metal NPs [53], which also revealed that M1 and M2 populations of macrophages remained unaffected.

### 2.4. Concentration of Pb in Blood Decreased after a 5-Week Clearance Period

**Figure 4:**
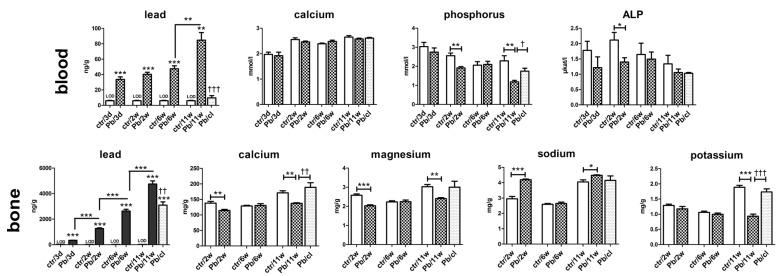
The concentrations of selected elements and alkaline phosphatase (ALP) in the blood and bone after Pb(NO_3_)_2_ NP inhalation. The graphs of selected elements (Pb, Ca, P, Mg, Na, K) and ALP in the blood and/or in the bone tissue. The graphs values indicate average ± SD for 5 mice/group; * *p* < 0.05, ** *p* < 0.01, *** *p* < 0.001 compared with the corresponding control group (ctr) or between adjacent time points, and † *p* < 0.05, †† *p* < 0.01, and ††† *p* < 0.001 compared with the corresponding Pb(NO_3_)_2_ NP group by unpaired *t*-test. The concentrations of Pb in controls were below limit of detection (LOD). LOD for Pb in the blood was 6, 11, 4 and 3 ng/g Pb at individual time points, for Pb in the bone 21 ng/g Pb.

The blood Pb level is a sensitive indicator of whole-body Pb exposure as it distributes the lead into secondary target tissues. Over recent decades, the United States Centers for Disease Control and Prevention (USCDC) has gradually lowered the blood Pb level at which medical intervention is necessary: From 60 μg/dL before 1975 to 10 μg/dL in 1991 [26]. The current limit recommended by the WHO is 5 μg/dL. In our experimental animals, the blood Pb concentration continuously increased as the exposure time to Pb(NO_3_)_2_ NPs increased (Figure 4, Table 3).

The concentration of Pb in blood increased slightly from 31 ng/g (3.1 μg/dL) after 3-day Pb(NO_3_)_2_ NP exposure, to 47 ng/g (4.7 μg/dL) after 6-week exposure, and to 85 ng/g (8.5 μg/dL) after 11-week exposure. Following a 5-week clearance period, such Pb concentration decreased approximately fivefold compared with the level in the 6-week Pb(NO_3_)_2_ NP exposure group (10 ng/g or 1 μg/dL).

Moreover, the exposure to inhaled Pb(NO_3_)_2_ NPs did not influence the blood level of Ca at any time point (Figure 4); however, the blood level of phosphorus (P) was affected (Figure 4). After 2 and 11 weeks of exposure to Pb(NO_3_)_2_ NP, the blood concentration of P was significantly decreased (*p* < 0.01) compared with control animals. The blood level of P in Pb/cl mice was similar to control animals.

ALP is an enzyme required to generate free P for hydroxyapatite formation during bone mineralization [54]. During hypophosphatemia (in our experiment, predominantly after 2 and 11 weeks of Pb(NO_3_)_2_ NP exposure), ALP activity could increase in order to compensate mineral dysregulation and to provide more phosphate to the bones [55]. However, the ALP level in mice exposed to Pb(NO_3_)_2_ NPs was decreased compared with control animals at all analyzed time points; this difference was only significant after 2 weeks of exposure due to high variability among animals. Nevertheless, the biological significance and consequences of low P levels accompanied by low ALP levels will require additional research.

### 2.5. A 5-Week Clearance Period Did Not Affect the Pb Level in Bones

Based on previous studies, the most of total body Pb burden occurs in the skeleton as the bone is the major target tissue for Pb storage [56,57]. The affinity of Pb for bone has been described predominantly after gastrointestinal exposure to Pb in solutions [58,59]. However, Pb can also persist for prolonged periods in the atmosphere due to its nature, representing thus the exposure route for humans [56]. Here, we analyzed mouse femurs as models of the axial skeleton. Only a few studies have analyzed the effect of inhaled Pb in the form of submicrometric particles on bone [60,61].

The concentration of Pb in femur was the highest among the studied organs (lung, liver, kidney and spleen) at all designated Pb(NO_3_)_2_ NP exposure times except for 3 days (Figure 4, Table 2). At this time, the Pb concentration was the highest in the kidney, followed by the femur (Table 2). The pattern of bone Pb content contrasted with the blood Pb level during Pb(NO_3_)_2_ NP inhalation. The increase of the Pb concentration in the inhaled air between 6 and 11 weeks corresponded with a rapid Pb increase in the blood, lungs, liver and kidneys (Table 2), but not in the bones.

The femur concentrations of Ca (*p* < 0.01) and Mg (*p* < 0.001) were significantly reduced after 2-week Pb(NO_3_)_2_ NP inhalation. Following 11-week Pb(NO_3_)_2_ NP inhalation, Ca (*p* < 0.01), Mg (*p* < 0.01) and K (*p* < 0.001) concentrations were significantly diminished in the femur compared with control animals (Figure 4). On the other hand, the Na level significantly increased in bones after 2- and 11-week Pb(NO_3_)_2_ NP exposure. This finding is in agreement with our previous study [24], which revealed the same trends in bone mineral content changes after inhalation of PbO NPs: Ca, Mg and K concentrations were significantly decreased, while Na was increased (not significantly). A 5-week clearance period increased the concentrations of Mg (not significantly), Ca (*p* < 0.01) and K (*p* < 0.001) in the bone relative to the corresponding control animal values. The effect of Pb(NO_3_)_2_ NP inhalation on femur weight (Figure S1) was similar to their alteration after exposure to PbO NPs [24]. There was only a small effect (slight reduction) in the Pb level in bones (femurs) after a 5-week clearance period (Figure 4).

### 2.6. Lead Is Stored in Ionic Forms in the Kidney Cortex after Pb(NO_3_)_2_ NP Exposure

**Figure 5:**
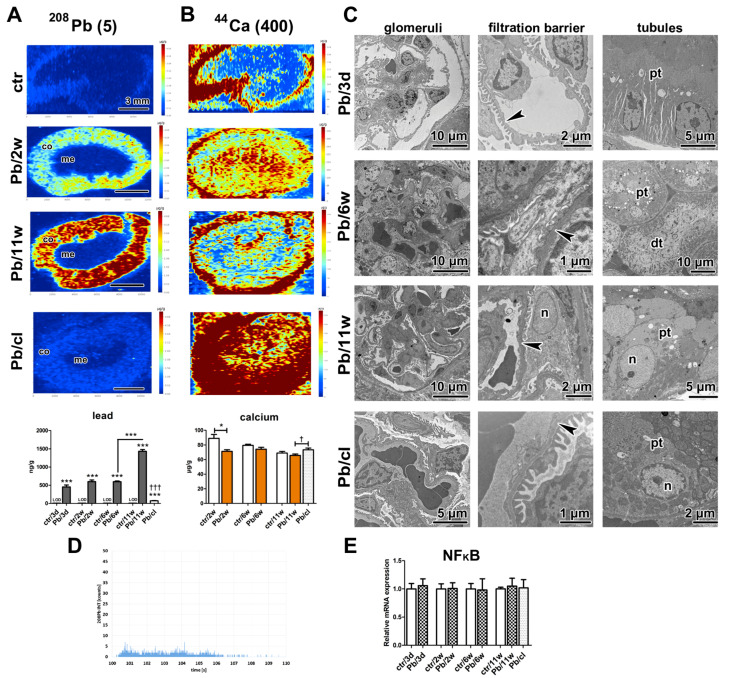
The effect of Pb(NO_3_)_2_ NP inhalation and its clearance on the kidney. (**A**) Distribution of Pb in kidney samples using laser ablation inductively coupled plasma mass spectrometry (LA-ICP-MS) after Pb(NO_3_)_2_ NP inhalation. The control kidney was without Pb positivity, Pb was detected in kidney cortex (co) after Pb(NO_3_)_2_ NP inhalation but not in the medulla (me). After a 5-week clearance period, Pb was still present in kidney cortex. The graph of Pb level in the kidney at designated time points. The graphs values denote average ± SD for 5 mice/group, *** *p* < 0.001 compared with the corresponding control group (or between adjacent time points), and ††† *p* < 0.001 compared with the corresponding Pb(NO_3_)_2_ NP group by unpaired *t*-test. Limit of detection (LOD) for Pb in the kidney was 9 ng/g. Number in parentheses shows maximal value (µg/g) of Pb on a scale. (**B**) Distribution of Ca in kidney samples using LA-ICP-MS after Pb(NO_3_)_2_ NP inhalation. The map of Ca exhibited higher distribution in kidney in Pb/cl group compared with the control and Pb(NO_3_)_2_ NP group. The graph of Ca level in the kidney at designated time points. Kidney Ca was significantly decreased after 2-week Pb(NO_3_)_2_ NP inhalation (* *p* < 0.05) compared with the corresponding control group (ctr) and significantly increased in Pb/cl group († *p* < 0.05) compared with the corresponding Pb(NO_3_)_2_ NP group. Number in parentheses shows maximal value (µg/g) of Ca on a scale. Scale bar in panels (**A**,**B**) = 3 mm. (**C**) Analysis of kidney ultrastructure in the transmission electron microscope (TEM). Proximal (pt) and distal tubules (dt) were without pathological changes up to 6-week Pb(NO_3_)_2_ NP inhalation. After 11-week Pb(NO_3_)_2_ NP inhalation, the proximal tubules of kidney exhibited changes both in nuclear (n) and cytoplasmic (c) architecture. A 5-week clearance period conducted to rescue of proximal tubule cells. The glomerular filtration membrane (GFM, arrowheads) was altered after 6-week Pb(NO_3_)_2_ NP inhalation. The nuclear alterations were observed also in nuclei (n) of podocyte (po) after 11-week Pb(NO_3_)_2_ NP inhalation. Scale bars are displayed individually for each picture. (**D**) Time-resolved signal originated from kidney tissue exposed to Pb(NO_3_)_2_ NPs. (**E**) Gene expression of *NF-κB* at selected time points after Pb(NO_3_)_2_ NP inhalation.

Next, we studied the kidneys because Pb is primarily excreted by this organ [62,63]. The concentration of Pb in kidneys continuously increased with the increasing length of exposure to Pb(NO_3_)_2_ NP (*p* < 0.001 at all time points; Figure 5A, Table 2).

As determined by both chemical and elemental analysis, upon 5-week clearance period, the level of Pb in kidneys was much lower compared to the state after 6 weeks of inhalation of Pb(NO_3_)_2_ NPs (*p* < 0.001, Figure 5A). Specifically, the level of Pb in kidneys decreased by about eightfold after 5-week inhalation of clean air (Table 2).

Elemental imaging revealed high Pb concentrations in the kidney cortex at all analyzed time points (Figure 5A). The kidney medulla exhibited no evident presence of Pb. A similar inhomogeneous distribution of Pb has been previously found in the kidney; however, the area with the highest Pb concentration was located more deeply in the kidney parenchyma (at the cortico-medullary junction) [64]. The levels of Pb in kidneys were, however, much higher both in so-called low (about eightfold) and high (fortyfold) conditions compared with our experiment, which could affect the Pb distribution in kidney tissue [64].

As the kidney cortex is the part of the imaged organs with the highest concentration of lead, we selected this area to confirm that Pb is present in the secondary target organ only in ionic form. For this purpose, different imaging mode was used (laser beam diameter was diminished, the signal of lead was monitored with the shortened integration time) and a small region of kidney cortex was analyzed (1.4 × 1.0 mm). The lead in nanoparticle form can be identified in this analysis by the presence of a high signal observed as yellow or red color in color-coded maps corresponding to sharp peaks on the graph displaying time-resolved signal. However, there were no Pb(NO_3_)_2_ NPs found in analyzed kidney cortex (Figure 5D) and the lead was present only in the ionic form, which was different from our previously published PbO NPs study, where distinct PbO NPs were identified [24].

The nephrotoxic effects of Pb are usually noted as high blood Pb concentrations > 50 μg/dL in children and >40 μg/dL in adults. Here, the blood Pb concentration reached rather low level of 8.5 μg/dL after 11-week Pb(NO_3_)_2_ NP exposure but we still observed morphological changes in kidney tissue at the ultrastructural level following the treatment. There were differences in the arrangement of filtration barrier in glomeruli of animals exposed to Pb(NO_3_)_2_ NPs (Figure 5C). The filtration membrane was diffusely thickened with intramembranous electron-dense deposits. Both *laminae rarae* were filled by electron-dense material, with a thicker *lamina densa* compared with controls. Pedicles (foot processes) of podocytes were irregularly arranged or lost after 11-week Pb(NO_3_)_2_ NP exposure.

Renal tubules (proximal and distal) displayed the typical physiological appearance even after 6-weeks of Pb(NO_3_)_2_ NP inhalation; however, after 11 weeks, there were obvious morphological alterations of the epithelial cells in proximal tubules. These findings are consistent with previously detected Pb nephrotoxicity that predominantly resulted in the damage of proximal renal tubules [62]. The apical parts of the epithelial cells were dilated, brush border was absent and cell organelles were randomly dispersed in the cytoplasm of some proximal tubules (Figure 5C). The cell nuclei were altered to euchromatin-rich structures, a change indicative of augmented transcriptional activity both in glomerular and tubular parts of the kidney after 11-week Pb(NO_3_)_2_ NP inhalation (Figure 5C). It is of note that there was evident regeneration of glomerular and tubular kidney tissue after the 5-week clearance period. Moreover, the level of *NF-κB* in kidneys remained unaffected even after 11-week exposure to Pb(NO_3_)_2_ NPs (Figure 5E).

The kidney participates in the maintenance of physiological homeostasis via regulation of the extracellular fluid volume, acid-base balance and electrolyte blood concentrations. Especially sodium, potassium and calcium regulation in the body relies on key physiological processes in the kidney. We have not observed any alterations in blood concentrations of Na and K in animals that were exposed to Pb(NO_3_)_2_ NPs (Table S3), the finding that documents the capability of kidney to maintain both the blood and kidney levels of these fundamental minerals in the physiological limits even after Pb(NO_3_)_2_ NP inhalation.

The sodium and potassium kidney concentrations determined by LA-ICP-MS were similar to those determined by atomic absorption spectrometry (AAS) (Figure S4). Our results are consistent with the previously published data on element distribution in mouse organs [65]. The potassium distribution was similar in the kidney parenchyma of the control, Pb(NO_3_)_2_ NP-treated and Pb/cl animals (Figure S4), with the strongest K positivity in the kidney medulla.

The blood level of Ca is typically maintained within a relatively narrow range. There are three main processes involved in the control of its level: Intestinal absorption, renal reabsorption and/or excretion and interchange between the bones and the blood [66]. Here, the blood levels of Ca were maintained constant in all experimental groups at each time points (Table S3). This finding demonstrates the ability of kidneys to maintain blood level of Ca level in physiological limits. Nevertheless, the levels of Ca in kidneys were changed upon Pb(NO_3_)_2_ NP inhalation. Previous studies have demonstrated an elevated Ca concentration in the kidney after exposure to Pb and/or Cd [67,68]. On the other hand, we previously reported decreased Ca levels after exposure to PbO NP [24]. In the present study, the Ca concentrations in kidneys (Figure 5B) also significantly decreased in Pb(NO_3_)_2_ NP-exposed animals in comparison to the controls, with a significant reduction after 2 weeks (*p* < 0.05). The lead may compete with Ca in kidney tubules and impaired Ca reabsorption can decrease the kidney Ca level; however, the precise mechanism that explains Ca behavior after metal exposure has not been uncovered yet.

### 2.7. Severe Pathological Changes Are Visible in Liver Even after a 5-Week Clearance Period

**Figure 6:**
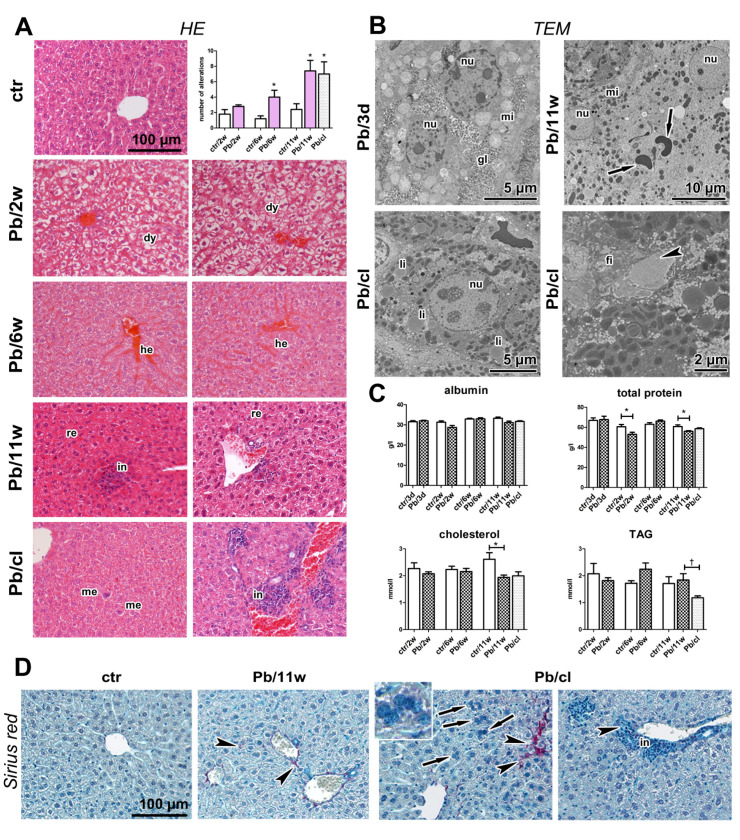
The effect of Pb(NO_3_)_2_ NP inhalation and its clearance on the liver. (**A**) The liver in control animals was without serious pathological changes (HE staining). The liver after 2, 6, and 11-weeks of Pb(NO_3_)_2_ NP inhalation exhibited hepatocyte dystrophy (dy), remodeling in liver parenchyma (re) and expressive hemostasis (he). We observed infiltrates (in) and megakaryocytes (me) in liver. Statistical evaluation of the histopathological changes in both control and Pb(NO_3_)_2_ NP-exposed groups in the experiments delineated according to Table S4. The graphs values denote average ± SD; * *p* < 0.05 compared with the corresponding control group (ctr) by unpaired *t*-test. (**B**) Ultrastructure of liver in TEM. Binucleated hepatocyte with regularly distributed organelles (mitochondria, mi) and glycogen (gl) in a 3-day Pb(NO_3_)_2_NP group. Enlarged binucleated hepatocyte with altered nuclei (nu), randomly distributed organelles, and free erythrocytes (arrows) in cytoplasm in an 11-week Pb(NO_3_)_2_NP group. Hepatocyte with physiological morphology of nucleus and regularly arranged organelles in Pb/cl; however, lipid droplets (li) present in increased number. Perisinusoidal fibroblast (fi) and accumulated collagen fibrils (arrowhead) in space of Disse in a Pb/cl group. Scale bars are displayed individually for each picture. (**C**) The blood levels of albumin, total protein, cholesterol, and triacylglycerols after Pb(NO_3_)_2_ NP inhalation. The graphs values denote average ± SD for 5 mice/group; * *p* < 0.05 compared with the corresponding control group (ctr), † *p* < 0.05 compared with the corresponding Pb(NO_3_)_2_ NP group by unpaired *t*-test. (**D**) Sirius Red staining of liver samples to detect collagen fibrils. After 11-week Pb(NO_3_)_2_ NP inhalation and in Pb/cl group, the collagen fibrils were observed around small blood vessels and inside liver parenchyma (arrowheads). Polynucleated hepatocytes were observed in Pb/cl group (inbox, arrows). Scale bar in panels (**A**,**D**) = 100 µm.

The liver represents the central organ involved in the metabolic processes leading to Pb intoxication. Our previous analyses uncovered toxic effects of insoluble metal NPs on liver cells [19,69]. The inhalation of highly soluble Pb(NO_3_)_2_ NPs caused serious remodeling of the liver parenchyma, hydropic degeneration of some hepatocytes, focal necrosis, hemostasis in small veins and liver sinusoids, and resulted in fewer small vessels (Figure 6A).

**Figure 7:**
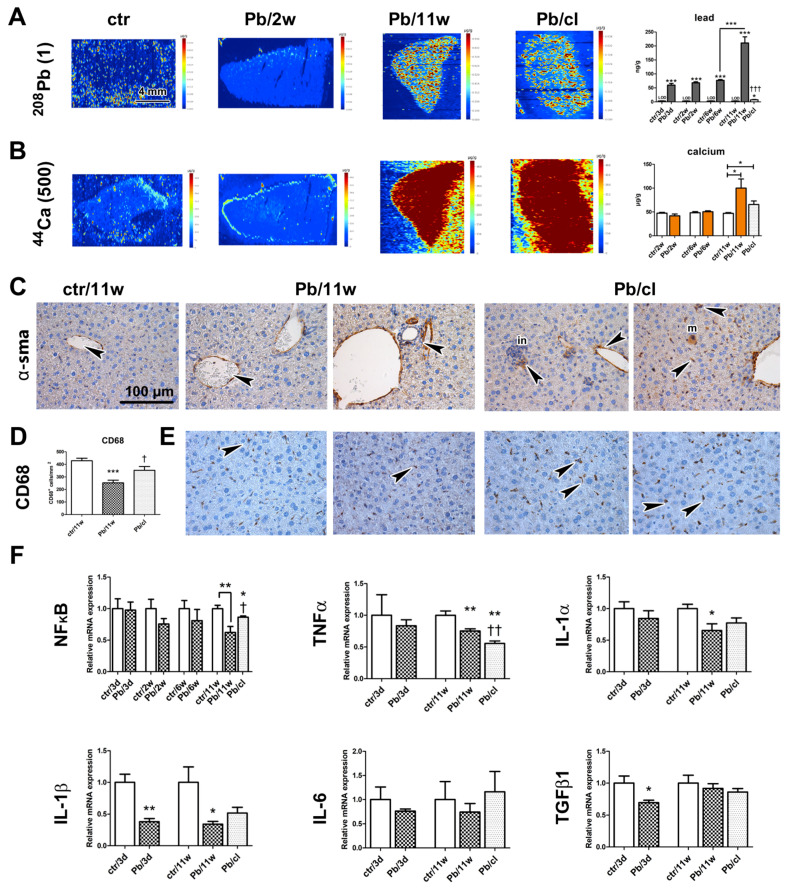
The distribution of selected metals at designated time points after Pb(NO_3_)_2_ NP inhalation in the liver, and immunohistochemical analyses of liver. (**A**) Distribution of Pb in liver samples using laser ablation and inductively coupled plasma mass spectrometry (LA-ICP-MS). The graph of Pb level in the liver at designated time points. (**B**) Distribution of Ca in liver samples using LA-ICP-MS. The graph of Ca level in the liver at designated time points. The graphs values denote average ± SD for 5 mice/group, * *p* < 0.05, *** *p* < 0.001 compared with the corresponding control group (or between adjacent time points), and ††† *p* < 0.001 compared with the corresponding Pb(NO3)2 NP group by unpaired *t*-test. LOD for Pb in the liver was 3 ng/g. Numbers in parentheses show maximal value (µg/g) of elements on a scale. Scale bar in panels (**A**,**B**) = 4 mm. (**C**) Immunohistochemical detection of alpha-smooth muscle actin (α-SMA) signal was located in the walls of blood vessels in control (arrowhead), and additionally α-SMA-positive cells were observed in liver parenchyma after Pb(NO_3_)_2_ NP inhalation (arrowheads), megakaryocyte (m) and focal infiltrate (in). (**D**) Statistical evaluation of the number of CD68-positive cells after Pb(NO_3_)_2_ NP inhalation. The graphs values denote average ± SD; *** *p* < 0.001 compared with the corresponding controls, and † *p* < 0.05 compared with the Pb(NO_3_)_2_ NP group by unpaired *t*-test. (**E**) Detection of CD68-positive cells (marker of macrophages) in liver (arrowheads). Scale bar in panels (**C**,**E**) = 100 µm. (**F**) Gene expression of nuclear factor κB (*NF-κB*) and cytokines at selected time points after Pb(NO_3_)_2_ NP inhalation. The graphs values denote average ± SD for 4-5 mice/group, * *p* < 0.05, ** *p* < 0.01 compared with the corresponding control group; † *p* < 0.05, †† *p* < 0.01 compared with the corresponding Pb(NO_3_)_2_ NP group by unpaired *t*-test.

We observed megakaryoblasts and megakaryocytes in the liver parenchyma. There were large inflammatory infiltrates in the parenchyma and the portal areas. There were also more hypertrophic hepatocytes and binucleated and polynucleated hepatocytes (up to seven nuclei) after Pb(NO_3_)_2_ NP inhalation; some hepatocyte nuclei contained large vacuoles. These histopathological changes in liver were statistically significant after 6 (*p* < 0.05) and 11 weeks (*p* < 0.05) of inhalation. There were also pathological changes in liver parenchyma after a 5-week clearance period; their number and extent were significantly greater in Pb/cl animals compared with control animals (*p* < 0.05). Interestingly, some Pb/cl animals displayed even more serious alterations than animals after 11 weeks of Pb(NO_3_)_2_ NP inhalation (Figure 6A, Table S4), although AAS analysis determined a marked decrease in the liver Pb after the clearance period (Figure 7A, Table 2).

Ultramicroscopic analyses uncovered the presence of numerous erythrocytes inside hepatocyte cytoplasm, alteration of hepatocyte nuclei to euchromatin rich, indicating increased transcriptional activity, and randomly dispersed cell organelles in the cytoplasm after 11 weeks of Pb(NO_3_)_2_ NP inhalation (Figure 6B). There was microvesicular steatosis in the Pb(NO_3_)_2_ NP–exposed and Pb/cl mice. Perisinusoidal fibroblasts exhibited high protein synthesis activity, and perisinusoidal space (of Disse) contained accumulated collagen fibrils (Figure 6B). Sirius Red staining revealed the presence of scattered collagen fibers in liver parenchyma (Figure 6D), predominantly after 6 and 11 weeks of Pb(NO_3_)_2_ NP exposure as well as in the clearance animals.

There were α-SMA-positive cells (Figure 7C) in the walls of hepatic blood vessels in the controls. In Pb(NO_3_)_2_ NP–exposed and Pb/cl mice, α-SMA-positive cells were also scattered in parenchyma or they were associated with cellular infiltrates. The number of hepatic macrophages (detected as CD-68-positive cells) was significantly decreased (*p* < 0.001) compared with the corresponding control animals after 11 weeks of Pb(NO_3_)_2_ NP inhalation (Figure 7D,E; Table S5). By contrast, there were more (*p* < 0.05) hepatic macrophages in Pb/cl animals compared with the corresponding Pb(NO_3_)_2_ NP animals.

Moreover, we observed some expression changes in liver on mRNA level. Downregulation of *NF-κB* was detectable already after 2 and 6 weeks and became statistically significant after 11-week exposure to Pb(NO_3_)_2_ NPs and the clearance period enabled for return of *NF-κB* expression close to its normal level (Figure 7F). Downregulation of *TNFα* occurred in the liver already after the shortest (3 days) as well as the longest (11 weeks) exposures (Figure 7F). Interestingly, the level of *TNFα* further decreased after a 5-week clearance period below its level at 11-week Pb(NO_3_)_2_ NP exposure (*p* < 0.01).

Also, *IL-1α* and *IL-1β* mRNA expressions were significantly downregulated after 3-day (*p* < 0.01 for *IL-1β*) and 11-week (*p* < 0.05 for both) exposures to Pb(NO_3_)_2_ NPs. The levels of *IL-1α* and *IL-1β* increased upon the 5-week clearance. The changes in the level of *IL-6* in the liver upon exposure to Pb(NO_3_)_2_ NPs were minimal and statistically insignificant.

The shortest (3 days) and the longest (11 weeks) exposures to Pb(NO_3_)_2_ NPs were also associated with significant downregulation in *TGFβ1* mRNA in the liver (*p* < 0.05) compared with the corresponding control animals (Figure 7F). The 5-week clearance did not cause any return of the *TGFβ1* mRNA towards its original level.

Pb(NO_3_)_2_ NP inhalation also caused expressive changes in blood biochemical parameters that manifested mainly as altered hepatic function (Table 4, Figure 6C). The level of total protein was significantly decreased after 2 (*p* < 0.05) and 11 (*p* < 0.05) weeks of Pb(NO_3_)_2_ NP inhalation. The albumin blood level, which also reflects liver function, was not significantly altered during the inhalation experiment (Table 4, Figure 6C). The association of the liver as the main organ involved in lipid metabolism was manifested in altered blood lipid levels. Total cholesterol (TCH) was significantly reduced (*p* < 0.05) in blood after 11 weeks of Pb(NO_3_)_2_ NP inhalation. Blood triacylglycerols (TAG) levels were increased after 6 and 11 weeks of Pb(NO_3_)_2_ NP inhalation compared with control animals. The TAG decrease was significant even after the clearance period (*p* < 0.05). Consistently, increased TAG levels after Pb exposure have been reported in rats that received Pb intragastrically [70] and in mice that received Pb orally [71]. However, not all reports have demonstrated the same trend in blood levels of TCH after Pb exposure [72]. One previous study described reduced TCH [70] similar to our findings, while another study described increased TCH levels [71]. Based on these findings, we conclude that sub-chronic exposure to a low level of Pb alters lipid homeostasis and affects thus blood lipid levels. This phenomenon, in turn, may disturb other tissues/organs in the organism.

The values of bilirubin and specific hepatic enzymes such as alanine aminotransferase (ALT), aspartate aminotransferase (AST) and gamma-glutamyl transferase (GGT) were in the physiological range after exposure to Pb (Table S3). Taken together, these data indicate that engineered highly soluble Pb(NO_3_)_2_ NPs used at a very low concentration cause persistent inflammatory changes in liver with impaired hepatic function.

The contents of Na and K in liver, as determined by AAS, were not altered at the analyzed time points (Figure S5). Interestingly, the Ca level after 11 weeks of Pb(NO_3_)_2_ NP inhalation was significantly increased (*p* < 0.05) compared with the corresponding control animals, while there was only a small difference in the Pb/cl animals (*p* < 0.05). We previously found a similar increase in Ca after 11 weeks of PbO NP inhalation [24]. Elemental imaging of liver samples revealed elevated Ca levels in Pb(NO_3_)_2_ NP-exposed and Pb/cl animals correspondingly to the quantitative AAS data (Figure 7B).

### 2.8. Comparison of Effects of Pb(NO_3_)_2_ NP and PbO NP Inhalation on Liver Parenchyma

The concentration of Pb in liver parenchyma continuously increased in parallel with lengthening of Pb(NO_3_)_2_ NP inhalation (*p* < 0.001, Figure 7, Table 2) and then decreased practically to the LOD after a 5-week clearance period (3 ng/g). The distribution of Pb in the liver parenchyma was practically homogeneous (Figure 7A).

When we compared the effects of highly soluble Pb(NO_3_)_2_ NPs applied in this study with the analogous effects observed previously upon application of less soluble PbO NPs [24] we came to the following conclusions: (1) While the total Pb concentrations in analyzed secondary organs in animals exposed to Pb(NO_3_)_2_ NPs were almost twofold lower compared with less soluble PbO NPs, the histopathological changes in liver were more serious than those caused by PbO NP inhalation. (2) A 5-week clearance period following Pb(NO_3_)_2_ NP exposure hardly promoted a positive effect on reparative processes in liver, although there was significant improvement and enhanced regeneration for a clearance period after PbO NP exposure. Obviously, higher solubility of Pb(NO_3_)_2_ NPs results in additional serious alteration of liver parenchyma compared to less soluble PbO NPs.

While the total Pb concentrations in lungs, kidneys and spleen in animals exposed to Pb(NO_3_)_2_ NPs (Table 2, Figure 5, Figure S6) was nearly twofold lower (corresponding with the inhaled mass concentration of Pb NPs) compared with less soluble PbO NPs [24], the liver Pb concentration was nearly fivefold lower compared with less soluble PbO NPs. Thus, the lead from inhaled Pb(NO_3_)_2_ NPs exhibited much lower propensity for liver accumulation than Pb contained in PbO NPs. This effect indeed requires further investigation since it could shed light on molecular processes involved in the initiation of reparative processes by more stable PbO NPs and/or their inhibition in the case of more soluble Pb particles.

## 3. Materials and Methods

### 3.1. Animals

Adult female mice (CD-1(ICR) BR strain) were obtained from the Animal Facility of the Masaryk University (Brno, Czech Republic). Females of ICR line were selected as they can be kept in larger groups and they are not aggressive. Animals were allowed to acclimatize to laboratory conditions for at least one week before the inhalation experiments. Commercial feeding and drinking water were provided ad libitum. The experiment was approved by the Ethical Board of the Institute of Analytical Chemistry, v.v.i., Czech Academy of Sciences, Brno (Approval No. 64/2016, 15 August 2016).

### 3.2. Generation of Pb(NO_3_)_2_ Nanoparticles

Lead(II) nitrate nanoparticles, (Pb(NO_3_)_2_ NPs), were generated continuously in situ in a home-made generator [74] by pneumatic atomization of 4 mM lead(II) nitrate solution (Lach_Ner, Neratovice, Czech Republic) in a nebulizer. The nebulizer was screwed in the wall of the atomizer (stainless steel tube, ID 14 mm, length 65 mm), which was oriented vertically. The solution was delivered to the nebulizer by means of a peristaltic pump (Ismatec, Glattbrugg-Zürich, Switzerland; type ISM 852; the flow rate 0.5 mL/min) was atomized at the nebulizer by the high-speed air jet (the flow rate of 12 L/min). The produced droplets were rapidly accelerated to the speed of the airstream and coarse droplets in the produced spray impacted the opposite wall of the atomizer and the formed liquid flows down due to gravity to the bottom from which it was continuously removed. The fine aerosol spray left the atomizer at the top and passed through a water trap, where large droplets were collected. The spray was then mixed in a drying chamber with dry filtered air (the flow rate of 3 L/min). Finally, Pb(NO_3_)_2_ NPs were diluted in the second step by a stream of air (20 L/min) and used for whole-body inhalation experiments.

### 3.3. Exposure to Pb(NO_3_)_2_ Nanoparticles

Mice treatment was performed in same inhalation chambers, the main characteristics of which we have been described previously [75]. Adult female mice with an average weight of approximately 24 g (about 6–8 weeks old) at the beginning of the inhalation experiment were continuously exposed to Pb(NO_3_)_2_ nanoparticles for 11 weeks (24/7). The food was sealed in a special box protected from deposition of nanoparticles from air. Control animals were exposed to the same air as treated animals without the addition of nanoparticles.

The samples from the controls and the Pb(NO_3_)_2_ NPs inhaled group of mice were collected at the designated time points of the inhalation period (three days, two weeks, six weeks). Ten biological replicates were harvested from the control (ctr/3d, ctr/2w, ctr/6w) and inhaling groups for each organ (Pb/3d, Pb/2w, Pb/6w). The lung, liver, kidney, spleen, femur and blood were collected and processed for chemical, biochemical, histopathological, ultramicroscopic, immunohistochemical, histochemical and immunofluorescent analyses, elemental imaging and to study the gene expression of selected markers.

Additionally, one group of mice inhaled air with Pb(NO_3_)_2_ NPs for six weeks (24/7) and then inhaled clean air for the next five weeks (this group is annotated as the clearance group Pb/cl). The 5-week clearance time point was selected to mirror our previous experiment with less soluble PbO nanoparticles [24]. This period was shown by us to be sufficient for reparation processes to be initiated at the end of the inhalation period (11 weeks). Ten biological replicates were harvested from the control (ctr/11w), Pb(NO_3_)_2_ NPs exposed (Pb/11w) and the clearance groups (Pb/cl), and selected organs were examined in the same way as previously collected samples of shorter time points.

### 3.4. Characterization of Generated Pb(NO_3_)_2_ Nanoparticles

Generated Pb(NO_3_)_2_ NPs were first characterized to obtain information about total future mice exposure (Table 1).

The size distribution of NP concerning particle count concentration was continuously measured directly inside the exposure cages using a Scanning Mobility Particle Sizer (SMPS; model 3936L72, TSI Inc., Shoreview, MN, USA). The average mass concentration of Pb(NO_3_)_2_ NPs was 68.6 µg Pb(NO_3_)_2_/m^3^ (i.e., 42.9 µg Pb/m^3^) during the inhalation experiment. Mass concentration of generated Pb(NO_3_)_2_ NPs was calculated by dividing the mass of Pb(NO_3_)_2_ NPs collected on the filter by the volume of the air sample that passed through the filter. Generated Pb(NO_3_)_2_ NPs were sampled on nitrocellulose filters (pore size 0.45 µm, diameter 25 mm, Millipore, Bedford, MA, USA) for 24 h, one filter per day. Filters were dissolved in HNO_3_ using a UniClever microwave mineralizer (Plazmatronika, Wroclaw, Poland) and Pb content in the sample was determined using AAS (AAnalyst 600, PerkinElmer Inc., Shelton, CT, USA).

Size and shape of the generated Pb(NO_3_)_2_ NPs were characterized by electron microscopy (EM). Immediately after generation at the generator output, Pb(NO_3_)_2_ NPs were collected by electrostatic precipitation using a Nanometer aerosol sampler (model 3089, TSI) on EM grids (copper S160-4, 3 mm in diameter, 400 mesh grids, Agar Scientific, Electron Technology, Stansted, Essex, UK). The samples were analyzed using a Magellan 400 L XHR microscope (FEI Company, Hillsboro, OR, USA), operating in the scanning transmission electron microscope (STEM) mode. The STEM analyses revealed that the Pb(NO_3_)_2_ NPs consist of spherical and irregular shaped bean-like formations without clear internal structure whose size correlates with the size obtained by SMPS.

The estimated deposited dose was calculated for pulmonary deposition fraction of 10% [76]. It corresponded to 0.774 µg of Pb(NO_3_)_2_ NPs (or 0.484 µg Pb) per gram of mouse body weight over the 11-week inhalation period, and 0.422 µg of Pb(NO_3_)_2_ NPs (or 0.264 µg Pb) per gram of mouse body weight for the clearance group over the 6-week inhalation period. Data used for calculation are shown in Supplementary Materials. The pulmonary deposition fraction calculated using the MPPD model [33,34,35] for the generated Pb(NO_3_)_2_ NPs (geometric mean diameter of 31.3 nm, mass concentration of 68.6 µg/m^3^, BALB/c strain, FRC of 0.30 mL, breathing frequency of 300 min^−1^, tidal volume of 0.2 mL, inspiratory fraction of 0.4) was 12.8%. This value is slightly higher than the value from the literature [76]. The corresponding deposition dose of Pb(NO_3_)_2_ calculated for DF = 0.128 was 0.991 µg of Pb(NO_3_)_2_ NPs (or 0.620 µg Pb) per gram of mouse body weight for 11 weeks and 0.540 µg of Pb(NO_3_)_2_ NPs (or 0.338 µg Pb) per gram of mouse body weight for 6-week inhalation period.

### 3.5. Histological Analysis

Samples of organs (lung, liver, kidney and spleen) designated for histological analyses were fixed overnight in 10% buffered neutral formaldehyde in a fridge and after that immersed in series of increasing concentrations of ethanol, xylene and paraffin wax. Serial histological sections of 5 µm thickness were prepared, and selected slices were stained by Hematoxylin–Eosin, and Sirius Red–Alcian Blue for analysis of collagen fibers. Toluidine Blue was used to detect mastocytes. The sections were examined by light microscopy in a blinded fashion by two histologists. We evaluated at least 8–10 slides per organ in 5 animals from the control group and 5 animals from the Pb-treated group after 2-week, 6-week and 11-week Pb(NO_3_)_2_ NP inhalation and assessed alterations in histopathological changes in whole sections (Tables S1 and S4).

Selected samples of lungs, liver, and kidneys were also immersed in 10% sucrose in PBS in a fridge overnight. Next day, samples were embedded into O.C.T. Compound (Agar Scientific Gardena, CA, USA), and stored at −25 °C for subsequent analysis. Further, the cryosections of 20 µm thickness of the lung, liver, and kidney samples were prepared, and used for LA-ICP-MS imaging.

Photos of the slices were taken by light microscope (Leica DM5000 B, Leica Microsystem GmbH, Vienna, Austria) and a digital color camera (Leica DFC480, Leica Microsystem GmbH, Vienna, Austria).

### 3.6. Immunohistochemistry

After deparaffinization and rehydration of the sections, citrate buffer (pH = 6) was used as a pre-treatment in a 97 °C water bath. For inhibition of non-specific secondary antibody binding, sections were incubated with a blocking serum (VECTASTAIN ABC Kit, Rabbit IgG, PK-4001, Vector Laboratories, USA; VECTASTAIN ABC Kit, Mouse IgG, PK-4002, Vector Laboratories, USA) for at least 20 min at room temperature (RT) and then, they were incubated with the primary antibody (α-SMA, CD68, MPO; detailed information Table S6).

After application of biotinylated secondary antibody (VECTASTAIN ABC Kit, Rabbit IgG, PK-4001, Vector Laboratories, Burlingame, CA, USA; VECTASTAIN ABC Kit, Mouse IgG, PK-4002, Vector Laboratories, Burlingame, CA, USA) for 40 min at RT, slices were incubated with the peroxidase-conjugated avidin-biotin complex (VECTASTAIN ABC Kit, Rabbit IgG, PK-4001, Vector Laboratories, Burlingame, CA, USA; VECTASTAIN ABC Kit, Mouse IgG, PK-4002, Vector Laboratories, Burlingame, CA, USA) for 30 min at RT. Chromogen substrate diaminobenzidine (Liquid DAB+ Substrate Chromogen System, K3468, DAKO, Carpinteria, CA, USA) was used for visualization of positive cells. Counterstaining of slices was performed by Hematoxylin. A negative control of the sample was obtained by omitting the primary antibody from the labeling protocol.

Photos were taken by light microscope (Leica DM5000 B, Leica Microsystem GmbH, Vienna, Austria) and a digital color camera (Leica DFC480, Leica Microsystem GmbH, Vienna, Austria). Number of macrophages in lungs and liver is presented as the mean ± SD. Analyses were performed on five mice per each group after 11-week inhalation (control group, Pb(NO_3_)_2_ NP group and Pb/cl group; Tables S2 and S5). The values of CD68+ macrophages were counted per square millimeter. Number of CD68+ macrophages was evaluated from two (liver) slides or four (lungs) slides (10 images/slide) in each animal. The total area of analyzed lungs was 3.346 mm^2^ per each animal and in case of liver it was 1.673 mm^2^ per each animal.

### 3.7. Immunofluorescence

After deparaffinization and rehydration, samples were pre-treated in citrate buffer (pH = 6) in a 97 °C water bath. Blocking serum (VECTASTAIN ABC Kit, Rabbit IgG, PK-4001, Vector Laboratories, Burlingame, CA, USA) was applied for at least 20 min at RT to inhibit non-specific secondary antibody bindings.

Then, samples were incubated with primary antibody for 1 h at RT (α-SMA; detailed information Table S6). Secondary antibody (Goat anti-Rabbit IgG (H+L), Alexa Fluor 488, R37116, Invitrogen, USA) was applied for 40 min at RT. Sections were covered with a coverslip in reagent containing DAPI (Fluoroshield with DAPI, D9564, Sigma-Aldrich, St. Louis, MI, USA). Photos were taken under a fluorescence microscope (Leica DM LB2, Leica Microsystems GmbH, Vienna, Austria) and a digital color camera (Leica DFC480, Leica Microsystems GmbH, Vienna, Austria).

### 3.8. Transmission Electron Microscopy (TEM)

Samples of lungs, livers and kidneys were fixed in 3% glutaraldehyde for 24 h, washed three times in 0.1 M cacodylate buffer and post-fixed in 1% OsO_4_ solution for 1.5 h. After washing in cacodylate buffer, all samples were dehydrated in a series of increasing concentrations of ethanol, followed by acetone and embedded in the epoxy resin Durcupan ACM followed by 3 days of polymerization. The ultrathin sections (about 60 nm thick) were prepared for TEM analysis. The sections were cut using an ultramicrotome Leica EM UC6 (Leica Microsystem GmbH, Vienna, Austria) and placed on formvar-coated nickel grids. Some sections were without further contrasting for analysis of nanoparticles in electron microscope, the others were contrasted with uranyl citrate and lead acetate. Minimally four electron microscopy grids with 3–5 sections on each were analyzed for all organ samples at designated time points. All sections were observed using Morgagni™ 268 TEM (FEI Company, Eindhoven, Netherlands) working at 80 kV, designated structures were measured using iTEM software. The photographs were taken using a Veleta *CCD* camera (Olympus, Münster, Germany).

### 3.9. Biochemical Analysis of Blood

Blood samples (5 animals for each group: The control group, the experimental group exposed to the Pb(NO_3_)_2_ NPs and the clearance group at each selected time points) were collected by cardiac puncture into 1 mL lithium heparin tubes (TAPVAL, Dispolab, Czech Republic). The blood samples were centrifuged at 1000× *g* for 15 min. Biochemical parameters were determined using a clinical chemistry analyzer, Abbott Architect c4000 (Abbott Laboratories, IL, USA), wet chemistry system.

### 3.10. Determination of Lead in Mouse Blood and Organs

Blood samples (5 animals from each analyzed group at each time point) were collected by cardiac puncture into 1 mL plastic Eppendorf tubes containing a small amount of heparin. Whole-blood (0.3–0.8 g) was divided by centrifugation into erythrocytes, white blood cells and plasma. After centrifugation, 300 µL of methanol was added to the fraction of white blood cells and the plasma (55% of mass of whole blood) and formed precipitate (plasma proteins, white blood cells etc.) was separated by another centrifugation from remaining supernatant. The separated samples were stored at 5 °C for subsequent analysis.

The weights of individual organs were determined, and the values were recorded for later quantitative evaluation.

The individual organs and blood fractions were decomposed by microwave (MW) assisted digestion in concentrated subboil grade (quartz distillation system model MSBQ 2, Maasen, Eningen, Germany) nitric acid, i.e., liver in 5 mL, lungs, spleen and kidneys in 3 mL, blood cells in 2 mL, femur and other blood fractions in 1 mL of acid. The samples were treated in pre-cleaned quartz tubes of a closed pressurized autoclave system (UltraWave, Milestone s.r.l., Sorisole, Italy). The decomposition program consisted of four steps: 1st step—10 min with temperature ramp between 100 and 120 °C; 2nd step—5 min with temperature ramp between 120 and 200 °C; 3rd step—3 min with temperature ramp between 200 and 250 °C; 4th step—5 min at 250 °C. After cooling down (a duration of approx. 10 min), digests were quantitatively transferred to high-density polyethylene vials, diluted and adjusted with ultrapure water (Ultra Clear system-Evoqua Water Technologies, Barsbüttel, Germany) to the final mass of 10 g for organs, 4 g for blood cells and 3 g for femur and other blood components, respectively. Simultaneously, blank samples (typically n = 30 per sampling series) were processed analogously. The detection limit (LOD) of the method was calculated as three times the standard deviation of process blanks. All samples as well as blanks were processed in a clean laboratory with flowboxes.

The content of lead in the digests was determined by electrothermal atomic absorption spectrometry (ET AAS) employing AAnalyst 600 PerkinElmer (Waltham, MA, USA) instrumentation under recommended conditions. A mixture of ammonium phosphate and magnesium nitrate was used as a combined chemical modifier. The method of standard addition calibration was applied for quantitation.

In determination of lead in blood and organ samples, QA/QC (quality assurance/quality control) was performed on the day to day basis by recovery tests, and on the long-term basis by running CRM (certified reference material) along with samples. CRM of Lead Standard for AAS (TraceCERT^®^, 1000 mg/L Pb in nitric acid, Sigma-Aldrich) was used for the calibration and standard additions to the samples.

### 3.11. Determination of Element Content (Na, K, Mg, Ca) in Mouse Organs

The content of basic element components (Na, K, and Ca) in mouse organs was determined in the same sample solution prepared for determination of lead concentration. After dilution of samples, the concentrations of Na, K, Mg and Ca were determined by flame atomic absorption spectrometry, employing ContrAA 300 Analytik Jena (Germany) High Resolution Continuum Source AAS instrumentation under recommended conditions, using acetylene-air flame (Na, K, Mg) and acetylene-nitrous oxide flame (Ca), respectively. Measurement was performed at prominent analytical lines (Na 589.0 nm, K 766.5 nm, Mg 285.2 nm and Ca 422.7 nm). Calibration was based on certified analyte standard solutions Astasol^®^ (1 ± 0.002 g/L) (Analytika Ltd. Prague, Czech Republic).

### 3.12. LA-ICP-MS Analysis

The cryosections of lungs, kidneys and livers were analyzed by Laser ablation with Mass spectrometry of inductively coupled plasma (LA-ICP-MS) to obtain elemental distribution in these sections. The LA-ICP-MS setup consists of a laser ablation system UP213 (NewWave Research, USA), and ICP-MS Agilent 7500ce (Agilent Technologies, Japan). The laser ablation of the samples was done with a laser spot diameter of 100 μm, scan speed of 200 μm/s, repetition rate of 10 Hz and a laser beam fluence of 3 J/cm^2^. The content of lead (Pb), sodium (Na), potassium (K), calcium (Ca), iron (Fe) and zinc (Zn) was quantified using a series of agarose gels doped with a known amount of Pb, Na, K, Ca, Fe and Zn.

Imaging of Pb in kidney tissue sections was performed under different LA-ICP-MS parameters than elemental imaging of tissues. For this purpose, the laser beam diameter was diminished to 20 µm and the scan speed was 200 μm/s. During this imaging, isotope ^208^Pb with the integration time of 1 ms was measured. The size of exposed kidney tissue samples was 1.0 × 1.4 mm.

### 3.13. qRT-PCR Analysis

Total RNA was extracted using the RNeasy Plus Mini Kit (Cat. No. 74136, Qiagen, Germantown, MD, USA). Complementary DNA was synthesized according to the manufacturer’s instructions using a gb Elite Reverse Transcription Kit (cat. No. 3012, Generi Biotech, CR). qRT-PCR was analyzed with a LightCycler^®^ 480 (Roche). The number of analyzed cDNA samples was n = 3–5 for each group.

TaqMan Gene Expression Assay (cat. No. 4351372, Applied Biosystems, USA) for *Nf-kB1* (ID: Mm00476361_m1), *Tgfβ1* (ID: Mm03024053_m1), *Il-6* (ID: Mm99999064_m1), *Il-1α* (ID: Mm00439620_m1), *Il-1β* (ID: Mm01336189_m1) and *Tnfα* (Mm_00443258_m1) were used and gene expression was analyzed with the following program: Initial activation step at 95 °C for 10 min, followed by 45 cycles at 95 °C for 15 s, annealing temperature at 62 °C for 60 s. Gene expression values for each sample were expressed in terms of the threshold cycle normalized to beta-actin (*Actb*; ID: Mm00607939_s1) expression.

### 3.14. Statistical Analysis

Statistical analyses were performed with GraphPad Prism 5 (GraphPad Software, Inc., La Jolla, CA, USA). Unpaired student *t*-test was used to determine differences between experimental and control groups. Results were reported as the mean value ± standard deviation. The values of *p* < 0.05 were considered to be statistically significant.

## 4. Conclusions

In summary, by combining the current data with previously published findings, we demonstrate that form (solubility) of metal nanoparticles determines on the final effects on the individual organs. Furthermore, we also demonstrated that the exposure to soluble Pb(NO_3_)_2_ NPs impairs functionality of immune system in both lungs and liver, and that many aspects of this loss prevent tissue recovery even after 5 weeks long clearance.

## Figures and Tables

**Table 1:** The characterization of generated Pb(NO_3_)_2_ NPs.

Parameter	Pb(NO_3_)_2_
Particle count concentration	1.94 × 10^5^ NPs/cm^3^
Surface area	1.68 × 10^3^ µm^2^/cm^3^
Mode	37.2 nm
Geometric mean diameter	31.3 nm
Geometric standard deviation	2.15
Mass concentration	68.6 µg/m^3^
Estimated deposited dose (after 11w)	0.774 µg/g

**Table 2:** The concentration of Pb (ng/g) in organs following Pb(NO_3_)_2_ NP inhalation determined by atomic absorption spectrometry (AAS).

		ctr/3d, 2w, 6w, 11w	Pb/3d	Pb/2w	Pb/6w	Pb/11w	Pb/cl
Lungs	Range		137–246	185–295	215–312	552–1130	
	Mean	<LOD *	184	239	262	752	<LOD *
	SD		40	39	47	225	
Femur	Range		282–372	1095–1423	2397–3120	4042–5576	2523–3941
	Mean	<LOD *	336	1269	2640	4750	3097
	SD		36	123	297	551	584
Kidney	Range		368–655	488–760	560–669	1259–1559	48–91
	Mean	<LOD *	454	599	597	1433	72
	SD		118	102	43	109	15
Liver	Range Mean SD	<LOD *	46–80	60–80	70–87	159–281	4–10
			60	68	77	211	7
			13	8	6	49	3
Spleen	Range				42–174	171–293	
	Mean	<LOD *	<LOD *	<LOD *	102	234	<LOD *
	SD				51	49	

Comparison of Pb concentration in lungs, femur, kidney, liver and spleen at different time points. * Limit of detection in lungs, femur, kidney, liver, and spleen was 26, 21, 9, 3, and 29 ng/g Pb, respectively. LOD—limit of detection.

**Table 3:** Pb concentration in blood determined by AAS.

		ctr/3d, 2w, 6w, 11w	Pb/3d	Pb/2w	Pb/6w	Pb/11w	Pb/cl
Erythrocytes (ng/g)	Range		54–92	63–92	72–114	93–213	10–43
	Mean	<LOD *	71	79	88	168	19
	SD		15	13	17	48	14
Precipitate (ng/g)	Range						
	Mean	<LOD *	<LOD *	<LOD *	<LOD *	<LOD *	<LOD *
	SD						
Supernatant (ng/g)	Range						
	Mean	<LOD *	<LOD *	<LOD *	<LOD *	<LOD *	<LOD *
	SD						
Blood (ng/g)		<LOD *	31	40	47	85	10
Blood (μg/dL)		<LOD *	3.1	4.0	4.7	8.5	1.0

The concentration of Pb in blood at different time points. * Limit of detection in erythrocytes in control animals, in precipitates (white blood cells, thrombocytes, proteins) at all data points, in supernatant (serum) at all data points and in the whole blood in control animals was 6, 11, 4 and 3 ng/g Pb, respectively.

**Table 4:** Blood biochemical analysis following Pb(NO_3_)_2_ NP inhalation.

		ctr/2w	Pb/2w	ctr/6w	Pb/6w	ctr/11w	Pb/11w	Pb/cl	CD-1 (ICR)
TP (g/L)	Range	56–66	46–59	58–67	63–69	57–62	54–58	56–60	53–60
	Mean	61	53 *	63	66	60	56 *	59	
	SD	5	5	4	3	3	2	2	
Alb (g/L)	Range	29–32	25–31	32–34	32–34	31–35	29–33	31–33	36–43
	Mean	31	29	33	33	33	31	32	
	SD	1	2	1	1	2	2	1	
Ca (mmol/L)	Range	2.44–2.74	2.35–2.56	2.30–2.47	2.31–2.59	2.50–2.80	2.51–2.68	2.54–2.67	2.48–2.88
	Mean	2.56	2.46	2.39	2.48	2.68	2.58	2.61	
	SD	0.14	0.08	0.06	0.11	0.13	0.06	0.06	
P (mmol/L)	Range	2.31–2.96	1.74–2.13	1.75–2.66	1.73–2.61	1.82–2.95	1.00–1.40	1.37–2.25	2.30–4.52
	Mean	2.56	1.92 **	2.16	2.10	2.40	1.18 **	1.74 †	
	SD	0.28	0.15	0.40	0.37	0.62	0.17	0.35	
ALP (µkat/L)	Range	1.8–2.9	1.1–1.8	0.6–2.7	0.8–2.0	1.17–2.27	0.7–1.4	1.0–1.1	0.8–2.2
	Mean	2.1	1.4 *	1.6	1.5	1.5	1.1	1.0	
	SD	0.5	0.3	0.8	0.5	0.5	0.2	0.1	
Urea (mmol/L)	Range	6.9–8.5	5.8–7.7	6.2–7.7	6.1–13.0	7.6–10.0	4.0–7.5	6.6–8.6	4.6–7.5
	Mean	7.8	6.9	7.2	9.2	8.6	5.9 *	7.5 †	
	SD	0.7	0.8	0.6	3.0	1.0	1.3	0.8	
Crea (mmol/L)	Range	33.7–40.3	35.1–40.4	29.5–36.5	31.9–32.9	33.5–39.5	28.8–35.4	33.1–34.8	26.5–35.4
	Mean	36.7	36.2	32.2	32.5	37.0	32.4 *	34.1 *	
	SD	2.8	3.8	2.8	0.4	2.5	2.8	0.6	
Chol (mmol/L)	Range	1.76–2.76	1.87–2.26	1.83–2.54	1.92–2.54	1.88–3.39	1.75–1.93	1.48–2.35	1.84–3.81
	Mean	2.27	2.07	2.23	2.16	2.66	1.94 *	2.00	
	SD	0.42	0.16	0.27	0.26	0.62	0.19	0.34	
TAG (mmol/L)	Range	1.55–3.18	1.48–2.09	1.41–1.99	1.73–3.11	1.05–2.38	1.14–2.49	0.94–1.34	0.27–2.44
	Mean	2.07	1.82	1.72	2.24	1.67	1.84	1.18 †	
	SD	0.77	0.24	0.21	0.51	0.64	0.52	0.16	

Data were obtained from five animals per every group. As reference values were used values of female mice crl:CD-1 (ICR) BR according to previously published data [73]. Reference biochemical values were converted to our used units. * *p* < 0.05, ** *p* < 0.01 compared with the corresponding control group (ctr) by unpaired *t*-test, † *p* < 0.05 compared with the corresponding Pb(NO_3_)_2_ NP group by unpaired *t*-test.

## Supplementary Materials

Supplementary Materials can be found at https://www.mdpi.com/1422-0067/21/22/8738/s1.
