# Supplementary material for: A Clearance Period after Soluble Lead Nanoparticle Inhalation Did Not Ameliorate the Negative Effects on Target Tissues Due to Decreased Immune Response
Source: Int J Mol Sci. 2020 Nov 19;21(22):8738. doi: 10.3390/ijms21228738 (PMC7699374; doi:10.3390/ijms21228738)
Supplement: Supplementary file 1 [file ijms-21-08738-s001.pdf]

International Journal of Molecular Sciences
MDPI

Supplementary Materials

A clearance period after soluble lead nanoparticle inhalation did not ameliorate the negative effects on target tissues due to decreased immune response

Jana Dumková [1], Tereza Smutná [1,2], Lucie Vrlíková [2], Bohumil Dočekal [3], Daniela Kristeková [2,4] Zbyněk Večeřa [3], Zuzana Husáková [5], Veronika Jakešová [2], Adriena Jedličková [2], Pavel Mikuška [3], Lukáš Alexa [3], Pavel Coufalík [3], Michaela Tvrdoňová [5], Kamil Křůmal [3], Tomáš Vaculovič [5], Viktor Kanický [5], Aleš Hampl [1] and Marcela Buchtová [2,4,*]

1 Department of Histology and Embryology, Faculty of Medicine, Masaryk University, 625 00 Brno, Czech Republic; jdumkova@med.muni.cz (J.D.); terka.smutna12@gmail.com (T.S.); ahampl@med.muni.cz (A.H.)
2 Laboratory of Molecular Morphogenesis, Institute of Animal Physiology and Genetics, v.v.i., Czech Academy of Sciences, 602 00 Brno, Czech Republic; vrlikova@iach.cz (L.V.); daniela.kristekova@gmail.com (D.K.); veronikam@volny.cz (V.J.); adri.jedlickova@gmail.com (A.J.)
3 Department of Environmental Analytical Chemistry, Institute of Analytical Chemistry, v.v.i., Czech Academy of Sciences, 602 00 Brno, Czech Republic; docekal@iach.cz (B.D.); vecera@iach.cz (Z.V.); mikuska@iach.cz (P.M.); alexa@iach.cz (L.A.); coufalik@iach.cz (P.C.); krumal@iach.cz (K.K.)
4 Section of Animal Physiology and Immunology, Department of Experimental Biology, Faculty of Science, Masaryk University, 625 00 Brno, Czech Republic
5 Department of Chemistry, Faculty of Science, Masaryk University, 625 00 Brno, Czech Republic; zuzka.husakova@seznam.cz (Z.H.); 358018@mail.muni.cz (M.T.); vaca_777@yahoo.com (T.V.); viktork@chemi.muni.cz (V.K.)
* Correspondence: buchtova@iach.cz

Received: date; Accepted: date; Published: date

Calculation of deposited dose of $Pb(NO_3)_2$ nanoparticles

The estimation of deposited dose was calculated based on previously published methodology [1, 2, 3] and based on the average mass concentration of $Pb(NO_3)_2$ nanoparticles (68.6 µg $Pb(NO_3)_2/m^3$).

Deposited dose=(C *RMV * T * DF)/BW [3]

Where C is average concentration in the exposure atmosphere 68.6 µg $Pb(NO_3)_2/m^3$ (68.6 ng $Pb(NO_3)_2$/L). RMV is respiratory minute volume (L/min) that can be calculated using the equation

RMV =0.499 * $BW^{0.809}$ L/min [1]. BW is average body weight (0.024 kg).

T is exposure time (min) equal to 110 880 min (11 × 7 × 24 × 60) for inhalation group (11 weeks) or 60 480 min (6 × 7 × 24 × 60) for clearance group (6 weeks), respectively.

DF is pulmonary deposition fraction (10%), therefore 0.1 [2]. Estimated deposition dose of $Pb(NO_3)_2$ was 0.774 µg per gram of mouse body weight over the 11 weeks inhalation period and 0.422 µg of $Pb(NO_3)_2$ NPs per gram of mouse body weight for the clearance group.

References:

[1] Bide, R. W., Armour, S. J., & Yee, E. (2000). Allometric respiration/body mass data for animals to be used for estimates of inhalation toxicity to young adult humans. *J Appl Toxicol, 20*(4), 273-290.

[2] Miller, F. J. (2000). Dosimetry of particles in laboratory animals and humans in relationship to issues surrounding lung overload and human health risk assessment: a critical review. *Inhal Toxicol, 12*(1-2), 19-57.

[3] Mitchell, L. A., Gao, J., Wal, R. V., Gigliotti, A., Burchiel, S. W., & McDonald, J. D. (2007). Pulmonary and systemic immune response to inhaled multiwalled carbon nanotubes. *Toxicol Sci, 100*(1), 203-214.

Table S1a: Lung – histopathological changes after 2-week $Pb(NO_3)_2$ NP inhalation

lung	2 weeks									
	co1	co2	co3	co4	co5	Pb1	Pb2	Pb3	Pb4	Pb5
bronchiolitis			++		+				+	+
thickened septa		++	+	++				+	++	++
hemorrhage			+					+		

Table S1b: Lung – histopathological changes after 6-week $Pb(NO_3)_2$ NP inhalation

lung	6 weeks									
	co1	co2	co3	co4	co5	Pb1	Pb2	Pb3	Pb4	Pb5
infiltrate perivasc.										+
bronchiolitis		+	+	+	++		+	+		+
thickened septa			+	++	++	+	++		++	+
alv. emphysema						+	+	+		+
hemorrhage										+
bronchiectasis							+		+	+

Table S1c: Lung – histopathological changes after 11-week $Pb(NO_3)_2$ NP inhalation

lung	11 weeks														
	co1	co2	co3	co4	co5	Pb1	Pb2	Pb3	Pb4	Pb5	cl1	cl2	cl3	cl4	cl5
inf. peribron.	+	+	+		+	+	+	+	+		+		+		
inf. perivasc.								+					+		
atelectasis									+	++			+		
bronchiolitis				+				+	+	+	++	+	+	+	+
thickened septa	+	+	++	+	+	+	+	+	+	++	++	+++	++	+++	++
alv. emphysema		+				+	++	+	+	+		+		++	
hemorrhage					+		+			+	+				+

We evaluated at least 8-10 slides per organ and assessed alterations in histopathological changes as follows: inflammatory cell infiltrate peribronchiolar, inflammatory cell infiltrate perivascular, atelectasis, bronchiolitis, thickened septa with congested capillaries, alveolar emphysema, hemorrhage, and bronchiectasis; co1 - co5 control animals, Pb1 - Pb5 exposed animals, cl1 - cl5 animals after a 5-week clearance period at designated time-points. The increased level of phenotype was labelled by increased number of + symbols, where "+" means mild phenotype, "++" moderate phenotype, and "+++" severe phenotype in relevant type of alteration in organ.

Table S2: Analysis of macrophage numbers in lungs

		number of macrophages/ slide	number of macrophages/ mm^2	mean number of macrophages/ mm^2

ctr/11w /1	range<br>mean<br>SD	16.0-18.6<br>**17.58**<br>1.11	210	**238.72**
ctr/11w /2	range<br>mean<br>SD	19.3-22.6<br>**20.40**<br>1.50	244	
ctr/11w /3	range<br>mean<br>SD	15.8-22.0<br>**19.33**<br>2.74	231	
ctr/11w /4	range<br>mean<br>SD	19.1-26.0<br>**22.75**<br>3.46	272	
ctr/11w /5	range<br>mean<br>SD	17.9-22.0<br>**19.80**<br>1.97	237	
Pb/11w/1	range<br>mean<br>SD	15.3-18.4<br>**16.85**<br>1.53	201	**178.77****
Pb/11w/2	range<br>mean<br>SD	13.3-18.0<br>**15.10**<br>2.01	181	
Pb/11w/3	range<br>mean<br>SD	13.6-15.1<br>**14.58**<br>0.68	174	
Pb/11w/4	range<br>mean<br>SD	12.6-19.6<br>**15.90**<br>2.89	190	
Pb/11w/5	range<br>mean<br>SD	11.5-13.2<br>**12.35**<br>0.87	148	
Pb/cl/1	range<br>mean<br>SD	14.9-20.2<br>**17.90**<br>2.25	214	**198.38***
Pb/cl/2	range<br>mean<br>SD	17.1-19.6<br>**18.28**<br>1.31	218	
Pb/cl/3	range<br>mean<br>SD	15.5-17.3<br>**16.50**<br>0.74	197	
Pb/cl/4	range<br>mean<br>SD	13.8-16.2<br>**15.33**<br>1.11	183	
Pb/cl/5	range<br>mean<br>SD	13.9-15.9<br>**14.98**<br>0.98	179	

Data are presented as mean ± SD; analyses were performed with five mice per each group. Number of CD68+ macrophages was evaluated from 4 slides (10 images/1 slide) of each animal. The values of CD68+ macrophages were counted per square millimeter; *$p < 0.05$, **$p < 0.01$ compared with the corresponding control group (ctr) by unpaired t-test.

Table S3: Blood biochemical analysis following $Pb(NO_3)_2$ NP inhalation

		ctr/2w	Pb/2w	ctr/6w	Pb/6w	ctr/11w	Pb/11w	Pb/cl	CD-1 (ICR)
Tbil (µmol/l)	range	3.2-3.6	2.4-2.6	2.6-3.7	2.1-3.3	3.3-4.3	2.9-5.4	3.4-3.9	2.7-4.6
	mean	3.4	3.9	3.3	2.7	3.8	3.9	3.7	
	SD	0.2	0.6	0.5	0.5	0.4	0.9	0.2	
ALT (µkat/l)	range	0.6-3.3	0.7-4.1	0.3-1.4	0.3-0.5	0.27-0.40	0.3-0.6	0.2-0.5	0.3-0.7
	mean	1.4	1.5	0.6	0.5	0.3	0.4	0.4	
	SD	1.3	1.4	0.4	0.1	0.1	0.1	0.1	
AST (µkat/l)	range	3.7-14.6	6.6-12.1	2.5-9.4	1.7-5.5	1.80-3.13	1.1-3.4	2.2-3.0	0.8-1.3
	mean	8.2	8.4	4.9	3.4	2.3	2.2	2.5	
	SD	4.6	2.3	3.1	1.4	0.6	0.9	0.3	
Na (mmol/l)	range	146-149	140-148	141-149	138-146	146-148	143-146	140-147	151-161
	mean	147	145	144	142	147	144	144	
	SD	1	3	3	3	1	1	3	
K (mmol/l)	range	6.1-8.2	7.0-10.8	5.8-8.9	6.6-9.2	5.2-6.9	5.1-7.4	6.7-9.5	8.1-12.2
	mean	7.3	8.0	7.2	8.1	6.2	6.3	7.9	
	SD	0.9	1.6	1.6	1.2	0.9	1.0	1.3	
Cl (mmol/l)	range	115-125	114-121	115-121	112-120	112-120	116-121	117-122	112-124
	mean	119	117	118	116	116	118	119	
	SD	4	2	2	3	3	2	3	
Glu (mmol/l)	range	8.2-11.3	8.8-11.3	8.4-13.8	7.3-14.0	8.0-12.0	8.1-11.6	10.1-12.1	8.0-17.8
	mean	10.2	10.7	11.0	11.0	9.7	10.0	11.0	
	SD	1.4	1.2	2.1	2.8	1.7	1.5	0.9	
GGT (µkat/l)	range	< 0.07	< 0.07	< 0.07	< 0.07	< 0.07	< 0.07	< 0.07	< 0.07

Data were obtained from five animals per every group. As reference values were used values of female mice crl:CD-1 (ICR) BR of different strains of female mice according to Serfilippi et al. (2003). Reference biochemical values were count to our used units.

Table S4a: Liver – histopathological changes after 2-week $Pb(NO_3)_2$ NP inhalation

liver	2 weeks									
	co1	co2	co3	co4	co5	Pb1	Pb2	Pb3	Pb4	Pb5
mononuclear cell inf.	+	+		++						
hemostasis		+	+			+		+	+	+
hepatic remodeling			+				+			
infiltrate in portal area		+		+						
hep. dystrophy						++	++	++	+	++

Table S4b: Liver – histopathological changes after 6-week $Pb(NO_3)_2$ NP inhalation

liver	6 weeks									
	co1	co2	co3	co4	co5	Pb1	Pb2	Pb3	Pb4	Pb5
mononuclear cell inf.	+	+	+				+			+
focal necrosis						+	+			
hemostase		+	+	+		++	++	+	+	+
hepatic remodeling						+	++	++		
infiltrate in portal area							+			+
hep. dystrophy						+			+	

Table S4c: Liver – histopathological changes after 11-week $Pb(NO_3)_2$ NP inhalation

liver	11 weeks														
	co1	co2	co3	co4	co5	Pb1	Pb2	Pb3	Pb4	Pb5	cl1	cl2	cl3	cl4	cl5
mononucl. cell inf.	+	+	+		+	+	+	+	+	+	++	+++	+	+	+
focal necrosis						+		+	+			+	+		+
polynuclear hep.									+	+		+			
steatosis macroves.										+					
hemostasis		+	+	+		++	+		++	+	++	+	++		++
hepatic remodeling		+				++	+	+	++	+	+	+	++		+
hypertrophic hep.		++		+		+	+		+						+
infiltrate in portal area				+		+		+	+	+	+	++	+		+
hep. dystrophy								+							
vacuoles in nuclei						+			+	+			+		+
megakaryocytes						+			+		+	+			+

We evaluated at least 8-10 slides per organ and assessed alterations in histopathological changes as follows: mononuclear cell infiltrate, focal necrosis (degenerating hepatocytes), polynuclear hepatocytes, macrovesicular steatosis, hemostasis, hepatic remodeling, hypertrophic hepatocytes, infiltrates in portal area, hepatocyte dystrophy, vacuoles in hepatocyte nuclei, presence of megakaryocytes; co1 - co5 control animals, Pb1 - Pb5 exposed animals, cl1 - cl5 animals after a 5-week clearance period at designated time-points.

The increased level of phenotype was labelled by increased number of + symbols, where "+" means mild phenotype, "++" moderate phenotype, and "+++" severe phenotype in relevant type of alteration in organ.

Table S5: Analysis of macrophage numbers in liver

		number of macrophages/ slide	number of macrophages/ mm^2	mean number of macrophages/ mm^2
ctr/11w /1	range<br>mean<br>SD	31.4-33.0<br>**32.20**<br>6.49	385	**428.68**
ctr/11w /2	range<br>mean<br>SD	36.7-38.6<br>**37.65**<br>5.20	450	
ctr/11w /3	range<br>mean<br>SD	39.3-43.8<br>**41.55**<br>9.68	497	
ctr/11w /4	range<br>mean<br>SD	34.5-35.3<br>**34.90**<br>6.20	417	
ctr/11w /5	range<br>mean<br>SD	29.1-36.9<br>**33.00**<br>7.57	394	
Pb/11w/1	range<br>mean<br>SD	22.2-23.7<br>**22.95**<br>4.84	274	**252.59***
Pb/11w/2	range<br>mean	15.8-18.2<br>**17.00**	203	

	SD	5.32		
Pb/11w/3	range<br>mean<br>SD	23.8-26.6<br>**25.20**<br>5.97	301	
Pb/11w/4	range<br>mean<br>SD	14.2-19.3<br>**16.75**<br>5.97	200	
Pb/11w/5	range<br>mean<br>SD	23.0-24.5<br>**23.75**<br>3.96	284	
Pb/cl/1	range<br>mean<br>SD	34.4-39.0<br>**36.70**<br>8.27	439	**352.65***
Pb/cl/2	range<br>mean<br>SD	29.3-39.0<br>**34.15**<br>8.40	408	
Pb/cl/3	range<br>mean<br>SD	24.6-30.0<br>**27.30**<br>6.53	326	
Pb/cl/4	range<br>mean<br>SD	21.8-25.1<br>**23.45**<br>7.24	280	
Pb/cl/5	range<br>mean<br>SD	24.5-27.3<br>**25.90**<br>9.41	310	

Data are presented as mean ± SD; analyses were performed with five mice per each group. Number of CD68+ cells was evaluated from 2 slides (10 images/1 slide) of each animal, the values of cells were counted per square millimeter; ***$p < 0.001$ compared with the control group, and *$p < 0.05$ compared with the $Pb(NO_3)_2$ NP group by unpaired t-test.

Table S6a: List of antibodies used for immunohistochemical analysis

Primary antibody	Company	Catalog no.	Host species	Organs	Dilution	Time/temperature
α-SMA	Abcam	ab5694	rabbit	lung, liver	1:100	60 min/RT
MPO	Abcam	ab9535	rabbit	lung	1:50	60 min/RT
CD68	Abcam	ab125212	rabbit	lung, liver	1:100	60 min/RT

Table S6b: List of antibodies used for indirect immunofluorescence

Primary antibody	Company	Catalog no.	Host species	Organs	Dilution	Time/temperature
α-SMA	Abcam	ab5694	rabbit	lung, liver	1:100	60 min/RT

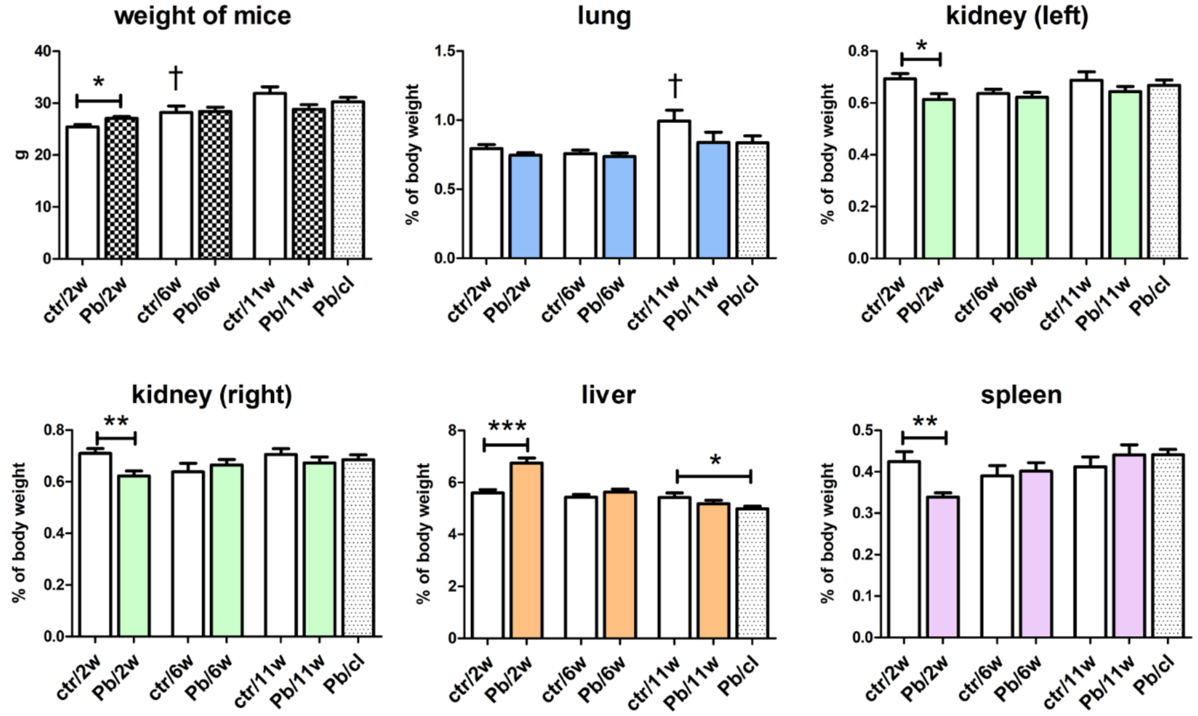

Figure S1. Weight of mice after $Pb(NO_3)_2$ NP inhalation. Lung, kidney, liver and spleen weight coefficient at different time points (2, 6 and 11 weeks).

The graphs values indicate average ± SD; * $p < 0.05$, ** $p < 0.01$, *** $p < 0.001$ compared with the corresponding control group (ctr), † $p < 0.05$ compared with the previous control by unpaired t-test.

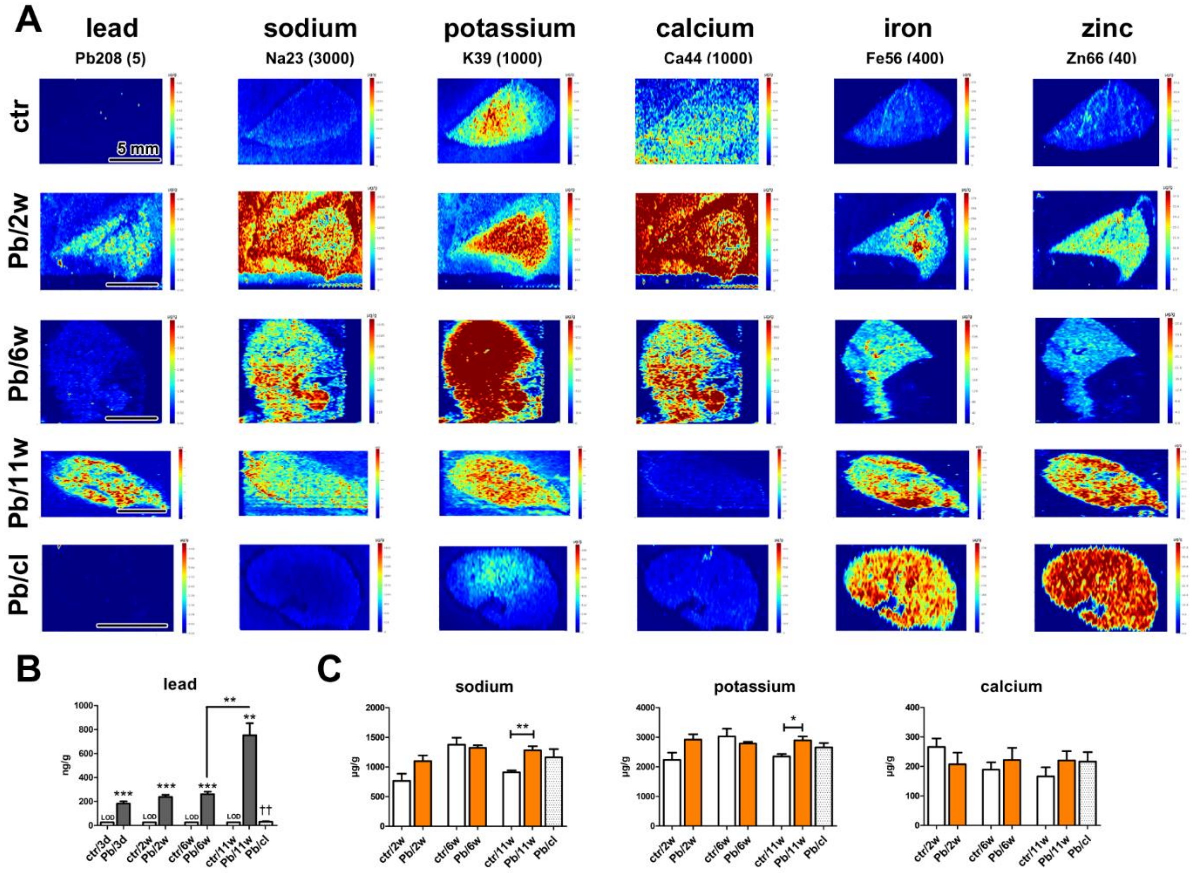

Figure S2. The distribution of selected metals at designated time points after $Pb(NO_3)_2$ NP inhalation in the lung.

A) Distribution of Pb, Na, K, Ca, Fe and Zn in lung samples using laser ablation inductively coupled plasma mass spectrometry (LA-ICP-MS). Numbers in parentheses show maximal value (μg/g) of element on a scale. Scale bar in all panels = 5 mm. **B**) The graph of Pb level in the lungs at designated time points. **C**) The graphs of Na, K and Ca level in the lungs at designated time points. The graphs values denote average ± SD for 5 mice/group, ** $p < 0.01$, *** $p < 0.001$ compared with the corresponding control group (or between adjacent time points), and †† $p < 0.01$ compared with the corresponding $Pb(NO_3)_2$ NP group by unpaired t-test. Limit of detection (LOD) for Pb in the lungs was 26 ng/g.

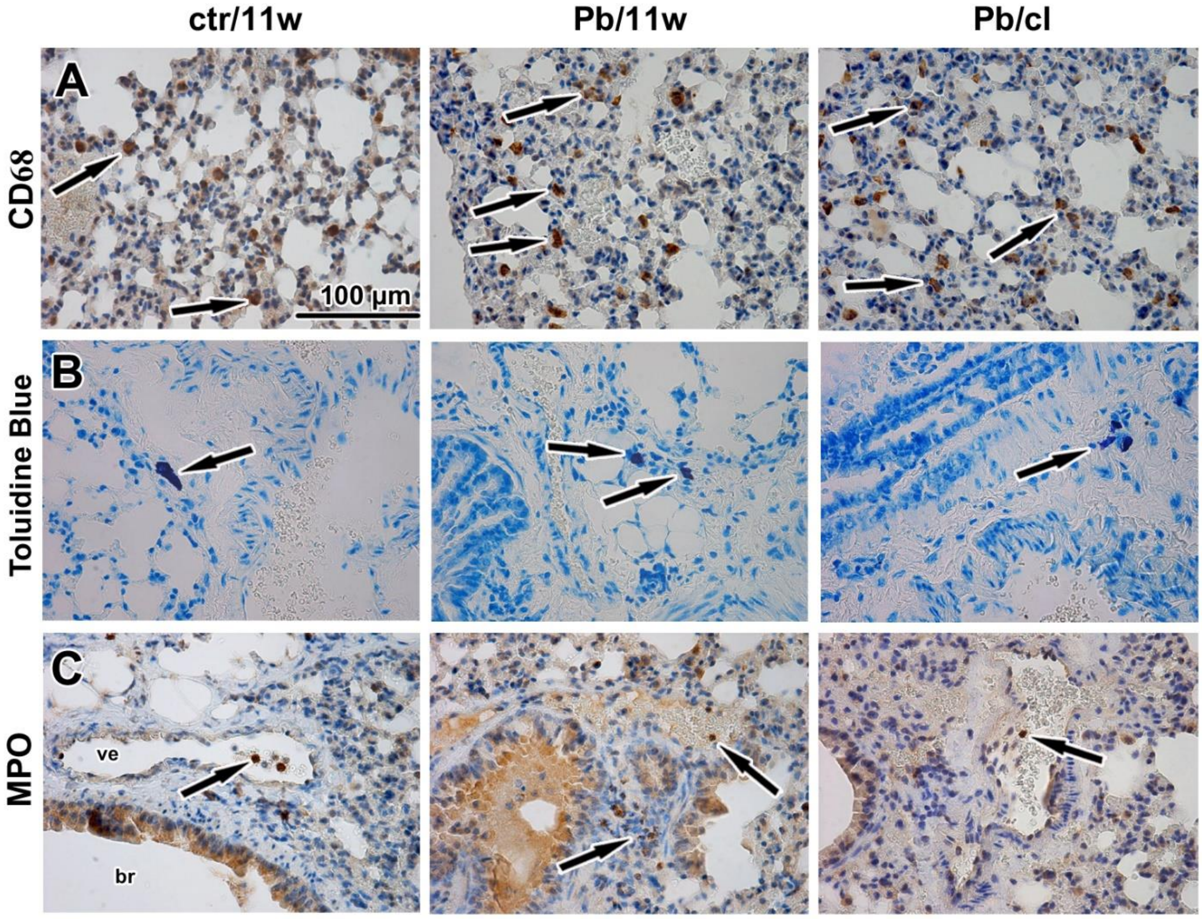

Figure S3: The effect of $Pb(NO_3)_2$ NP inhalation and its clearance on the lung inflammation.
A) Detection of CD68-positive cells (marker of macrophages) in lungs (arrows). **B**) Detection of Toluidine Blue-positive cells (marker of mastocytes) in lungs (arrows). **C**) Detection of MPO-positive cells (myeloperoxidase, marker of neutrophils) in blood vessels or in lung infiltrates in lung samples (arrows).

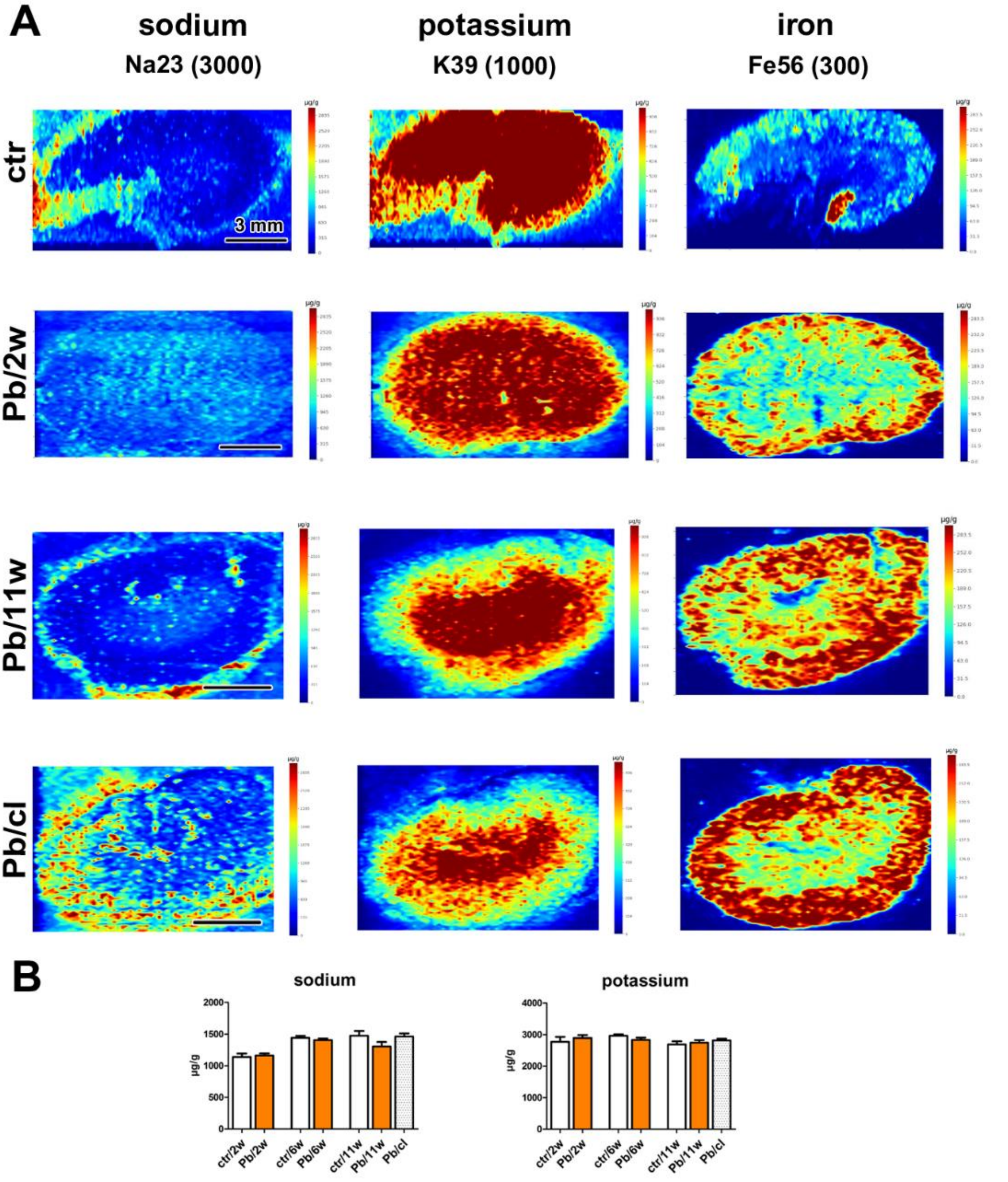

Figure S4. The distribution of selected metals at designated time points after $Pb(NO_3)_2$ NP inhalation in the kidney.

A) Distribution of selected elements (Na, K and Fe) in kidney samples using LA-ICP-MS after $Pb(NO_3)_2$ NP inhalation. Na and K were observed in similar manner in control and $Pb(NO_3)_2$ NP-exposed kidneys. The extent of the Fe was slightly increased after $Pb(NO_3)_2$ NP inhalation compared to the control. Numbers in parentheses show maximal value (µg/g) of element on a scale. Scale bar in all panels = 3 mm. **B**) The graphs of Na and K in kidney at designated time points. The graph values denote average ± SD for 5 mice/group.

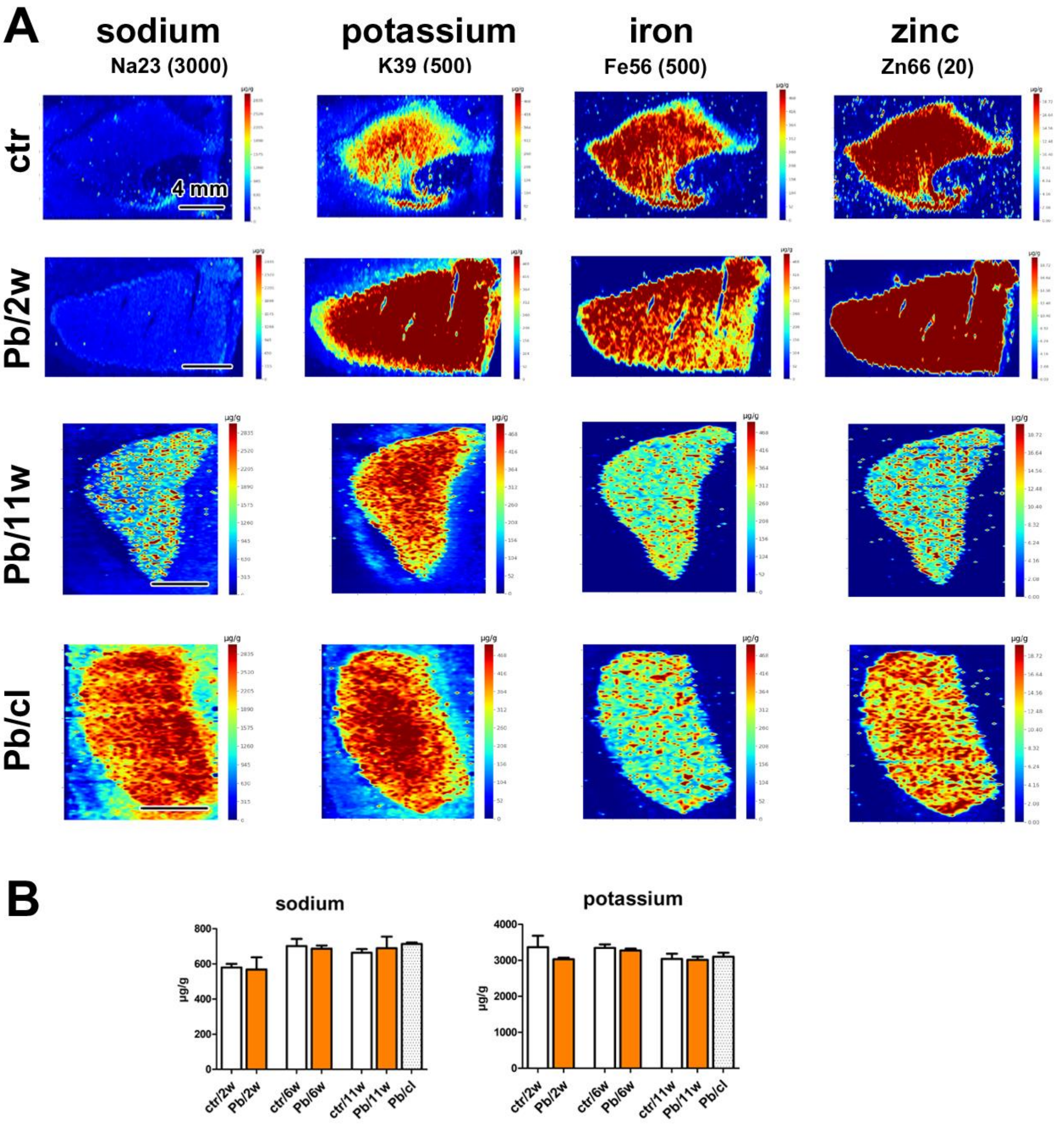

Figure S5. The distribution of selected metals at designated time points after $Pb(NO_3)_2$ NP inhalation in the liver.

A) Distribution of selected elements (Na, K, Fe and Zn) in liver samples using LA-ICP-MS after $Pb(NO_3)_2$ NP inhalation. Numbers in parentheses show maximal value (μg/g) of element on a scale. Scale bar in all panels = 4 mm. **B**) The graphs of Na and K in liver at designated time points. The graph values denote average ± SD for 5 mice/group.

Figure S6. The graph of Pb level in the spleen.

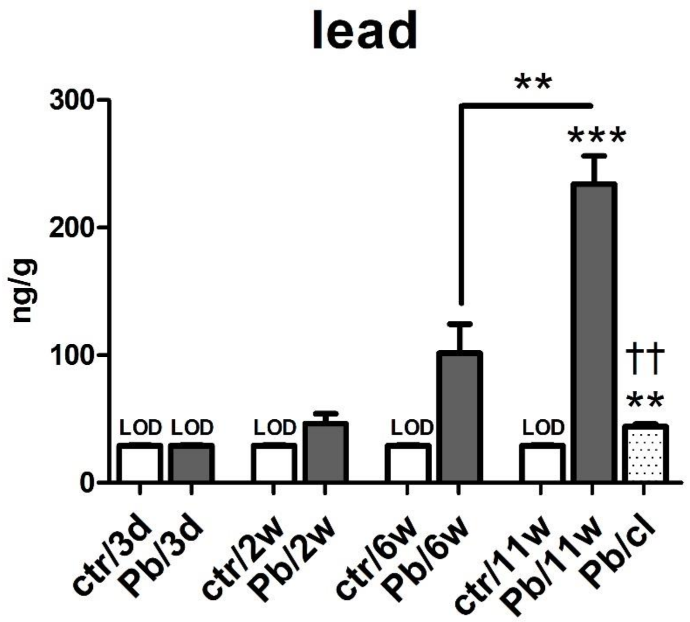

The graph of Pb level in the spleen at designated time points. The graphs values denote average ± SD for 5 mice/group, ** $p < 0.01$, *** $p < 0.001$ compared with the corresponding control group (or between adjacent time points), and †† $p < 0.01$ compared with the corresponding $Pb(NO_3)_2$ NP group by unpaired t-test. Limit of detection (LOD) for Pb in the spleen was 29 ng/g.

Publisher's Note: MDPI stays neutral with regard to jurisdictional claims in published maps and institutional affiliations.

© 2020 by the authors. Submitted for possible open access publication under the terms and conditions of the Creative Commons Attribution (CC BY) license (http://creativecommons.org/licenses/by/4.0/).
